# Differential regulation of serum microRNA expression by HNF1β and HNF1α transcription factors

**DOI:** 10.1007/s00125-016-3945-0

**Published:** 2016-04-08

**Authors:** Wojciech Fendler, Joanna Madzio, Kamil Kozinski, Kashyap Patel, Justyna Janikiewicz, Magdalena Szopa, Adam Tracz, Maciej Borowiec, Przemyslawa Jarosz-Chobot, Malgorzata Mysliwiec, Agnieszka Szadkowska, Andrew T. Hattersley, Sian Ellard, Maciej T. Malecki, Agnieszka Dobrzyn, Wojciech Mlynarski

**Affiliations:** Department of Paediatrics, Oncology, Haematology and Diabetology, Medical University of Lodz, Lodz, Poland; Department of Biostatistics and Translational Medicine, Medical University of Lodz, 36/50 Sporna Str., 91-738 Lodz, Poland; Studies in Molecular Medicine, Medical University of Warsaw, Warsaw, Poland; Laboratory of Cell Signalling and Metabolic Disorders, Nencki Institute of Experimental Medicine, Polish Academy of Sciences, Warsaw, Poland; Institute of Biomedical and Clinical Science, University of Exeter Medical School, Exeter, UK; Department of Metabolic Diseases, Jagiellonian University Medical College, Krakow, Poland; University Hospital, Krakow, Poland; Department of Clinical Genetics, Medical University of Lodz, Lodz, Poland; Department of Paediatric Diabetology, Medical University of Silesia, Katowice, Poland; Department of Paediatrics, Diabetology and Endocrinology, Medical University of Gdansk, Gdansk, Poland

**Keywords:** HNF, microRNA, MODY, Monogenic diabetes, Transcription factors

## Abstract

**Aims/hypothesis:**

We aimed to identify microRNAs (miRNAs) under transcriptional control of the HNF1β transcription factor, and investigate whether its effect manifests in serum.

**Methods:**

The Polish cohort (*N* = 60) consisted of 11 patients with *HNF1B*-MODY, 17 with *HNF1A*-MODY, 13 with *GCK*-MODY, an HbA_1c_-matched type 1 diabetic group (*n* = 9) and ten healthy controls. Replication was performed in 61 clinically-matched British patients mirroring the groups in the Polish cohort. The Polish cohort underwent miRNA serum level profiling with quantitative real-time PCR (qPCR) arrays to identify differentially expressed miRNAs. Validation was performed using qPCR. To determine whether serum content reflects alterations at a cellular level, we quantified miRNA levels in a human hepatocyte cell line (HepG2) with small interfering RNA knockdowns of HNF1α or HNF1β.

**Results:**

Significant differences (adjusted *p* < 0.05) were noted for 11 miRNAs. Five of them differed between *HNF1A*-MODY and *HNF1B*-MODY, and, amongst those, four (miR-24, miR-27b, miR-223 and miR-199a) showed *HNF1B*-MODY-specific expression levels in the replication group. In all four cases the miRNA expression level was lower in *HNF1B*-MODY than in all other tested groups. Areas under the receiver operating characteristic curves ranged from 0.79 to 0.86, with sensitivity and specificity reaching 91.7% (miR-24) and 82.1% (miR-199a), respectively. The cellular expression pattern of miRNA was consistent with serum levels, as all were significantly higher in HNF1α- than in HNF1β-deficient HepG2 cells.

**Conclusions/interpretation:**

We have shown that expression of specific miRNAs depends on HNF1β function. The impact of HNF1β deficiency was evidenced at serum level, making HNF1β-dependent miRNAs potentially applicable in the diagnosis of *HNF1B*-MODY.

**Electronic supplementary material:**

The online version of this article (doi:10.1007/s00125-016-3945-0) contains peer-reviewed but unedited supplementary material, which is available to authorised users.

## Introduction

Monogenic diabetes is a heterogeneous group of diseases caused by loss of function of single genes important for pancreatic beta cell function, survival or peripheral insulin action [1, 2]. Whereas each of the monogenic defects is fairly uncommon, together they are aetiological factors in potentially up to 2% of patients with diabetes, amounting to a prevalence of ~110 per million [3, 4]. *HNF1B*-MODY (or MODY5 [5]), is caused by dominant mutations of the gene encoding hepatocyte nuclear factor 1 beta (HNF1β), a POU-family transcription factor (TF) necessary for proper development of pancreatic beta cells and kidneys [6]. We hypothesised that hepatocyte nuclear factor 1 alpha (HNF1α) and HNF1β, as two structurally similar TFs, may regulate not only different genes, as shown in earlier gene expression studies [7], but also microRNAs (miRNAs). Expression of miRNAs is regulated by TFs, either indirectly as passengers of stimulated gene expression (in the case of miRNA sequences located within introns) or directly through TF binding to *cis* regulatory regions [8]. Experimental data in a mouse model of HNF1β knockdown presented by Kornfeld et al [9] showed that expression of multiple hepatic miRNAs depends on HNF1β function. If miRNA expression profiles vary at cellular levels, one could assume that their serum levels are also altered, as serum miRNAs originate from active secretion by cells or as a consequence of cell death [10]. This prompted us to search for an miRNA whose expression would be specifically altered in *HNF1B*-MODY. If indeed an *HNF1B*-MODY-specific miRNA expression pattern could be identified, it could bolster the current diagnostic strategies of monogenic diabetes, which involve the application of statistical tools to stratify candidates for genetic screening [11, 12], or biomarkers [13–15]. The objective of our study was to identify miRNAs under the transcriptional control of HNF1β or HNF1α and assess the potential of such miRNAs as biomarkers of monogenic diabetes.

## Methods

The study was approved by the Institutional Bioethics Committee at the Medical University of Lodz (no. RNN/18/12/KE 2012). All patients gave their informed consent for participation in the study; for children, parental approval was obtained instead.

## Group recruitment

The studied population consisted of two groups: the primary group and the replication group. Patients in the primary group were recruited from the Polish Registry of Monogenic Diabetes [4, 16]. The primary group was planned to include all known Polish patients with *HNF1B*-MODY with available serum samples, and four comparative groups: *HNF1A*-MODY, *GCK*-MODY (involving the gene encoding glucokinase [GCK]), type 1 diabetes and healthy controls. The two *HNF1*-MODY groups consisted of: 11 patients with *HNF1B*-MODY and 17 patients with *HNF1A*-MODY matched (1:1.5 ratio) for age, sex, BMI and HbA_1c_ levels. Three control groups were used: ten age- and sex-matched healthy individuals, 13 age- and sex-matched patients with *GCK*-MODY (to control for hyperglycaemia with preserved pancreatic islet beta cells) and nine HbA_1c_-matched patients with type 1 diabetes positive for at least two autoantibodies directed against pancreatic beta cells, and undetectable C-peptide levels at onset of diabetes.

The UK group (replication group) was recruited from the UK MODY registry [3] overseen by the Diabetes Research department and the Centre for Molecular Genetics at the University of Exeter Medical School and Royal Devon and Exeter Hospital. We selected patients with *HNF1B*-MODY with available serum samples, a matched *HNF1A*-MODY group and the three other comparative groups matched as above. Details about causative mutations in the three MODY groups of Polish and UK patients are provided in Table 1 of the electronic supplementary material (ESM).

## Molecular methods

### Serum miRNA profiling and measurements

Fasting serum samples were used in the study. We used real-time PCR arrays with locked nucleic acid-containing primers (miRCURY LNA; Exiqon, Copenhagen, Denmark) for serum profiling, as our hypothesis assumed a drop in miRNA expression, which necessitated high, detectable levels in healthy individuals. Exiqon real-time PCR human miRNA arrays A and B quantify expression of 752 miRNAs detectable in serum and provide very high sensitivity and reproducibility in comparison with other methods [17]. A standard protocol for circulating miRNA extraction, purification and analysis was used as in our previous study on circulating miRNA biomarkers [18]. In the replication group we quantified expression of miRNAs that were shown to be differentially expressed in the primary group. Quantitative real-time PCR (qPCR) was performed using Exiqon’s PCR miRNA-specific primer sets.

The reference miRNA for normalisation of the profiling assay was selected using the NormFinder algorithm [19]; the most stable reference was found to be the average expression level of assays detected in all samples. Thirteen such miRNAs were identified and their average threshold cycles (C_t_) were used as the reference for normalisation performed using the formula ∆${\mathrm{C}}_{\mathrm{t}}={\mathrm{C}}_{\mathrm{t}}\left(\mathrm{reference}\right)-{\mathrm{C}}_{\mathrm{t}}\left(\mathrm{miRNA}\;\mathrm{of}\;\mathrm{interest}\right)$, which provides a higher value for higher miRNA expression, facilitating its use and interpretation as a biomarker. For the replication group, we used the average expression of the three miRNAs (miR-142-3p, miR-126-3p, miR-16-5p) for normalisation. These miRNAs were selected out of the 13 used for normalisation in the primary experiment on the basis of universal and stable transcription and lack of significant differences between the five compared groups. A re-analysis of the primary group data with normalisation towards these three miRNAs yielded convergent results to normalisation against average expression level (data not shown). Details on isolation, reverse transcription and qPCR reactions in the primary group are shown in ESM Methods/Serum miRNA extraction, profiling and measurements. Details about the miRNA expression study in the replication group are shown in ESM Methods/miRNA real-time qPCR assays in the replication group.

### Silencing of *HNF1B* and *HNF1A*: cell line experiment

A human hepatocyte (HepG2) cell line was grown in an incubator at 37°C in 5% CO_2_ in DMEM (Sigma-Aldrich, Taufkirchen, Germany), supplemented with 10% fetal bovine serum (10270; Gibco, Darmstadt, Germany), 2 mmol/l l-glutamine (Gibco), 1 mmol/l sodium pyruvate (Sigma-Aldrich), 100 U/ml penicillin, and 100 μg/ml streptomycin (Gibco), until 80–90% confluence was reached. For silencing of *HNF1A* and *HNF1B* expression, the following small interfering RNAs (siRNAs) were used (all from Ambion, Austin, TX, USA): siRNA specific for HNF1α—s13868 (siRNA1a), s13869 (siRNA2a), s13870 (siRNA3a); siRNA specific for HNF1β—s13871 (siRNA1b), s13872 (siRNA2b).

A Nucleofector technology system (Amaxa Cell Line Nucleofector VCA-1003; Lonza, Basel, Switzerland) was used to transfect siRNA into the HepG2 cells; details on the procedure are listed in ESM Methods/Silencing of *HNF1B* and *HNF1A* genes—cell line experiment.

## Expression of *HNF1A* and *HNF1B* and HNF-dependent miRNAs

Total RNA was isolated from HepG2 cell pellet (10^6^ cells) using an RNeasy Mini Kit (Qiagen, Hilden, Germany) according to the manufacturer’s protocol. Coding DNA (cDNA; reverse-transcribed from RNA) was synthesised with High Capacity cDNA Reverse Transcription Kits (Applied Biosystems, Foster City, CA, USA). *HNF1A* and *HNF1B* mRNA levels were quantified using qPCR with the Mx3005P QPCR System (Agilent Technologies, Santa Clara, CA, USA). Western blots were used to confirm efficient HNF1α and HNF1β silencing at the protein level. Expression of miRNAs differentially expressed in *HNF1A*- and *HNF1B*-MODY was quantified using qPCR in cell lysate and culture medium. Measurement of miRNA expression was done similarly to the serum part of the study using TaqMan miRNA Assays (Applied Biosystems). All reactions were run in triplicate. U6 small nuclear RNA was used as an endogenous control (Applied Biosystems). In-depth description of molecular methods is provided in ESM Methods/Expression of *HNF1A* and *HNF1B* and HNF-dependent miRNAs.

## Statistical analysis

We used linear discriminant analysis for initial group separation. One-way ANOVA was performed to screen miRNAs for those showing differential expression between the tested groups. The Benjamini–Hochberg correction was used to control the false discovery rate. All miRNAs with a *p* value and false discovery rate below 0.05 were compared using Tukey’s post hoc test to confirm between-group differences. Covariate-adjusted comparisons in the UK group were performed using analysis of covariance (ANCOVA) with Tukey’s post hoc test. These comparisons were adjusted for patients’ sex, age, BMI and HbA_1c_ levels. A *p* value below 0.05 was considered statistically significant. Categorical variables were compared using the χ^2^ test. Statistica version 12.5 (StatSoft, Tulsa, OK, USA) and MultiExperiment Viewer (Dana Farber Cancer Institute, Boston, MA, USA) were used for statistical analyses. Receiver operating characteristic curves were created for differentially expressed miRNA that replicated in both cohorts. A multivariate classifier model was created using logistic regression. In order to retain the full sample size of the multivariate model, in the primary group missing expression values were imputed with average expression levels of a specific miRNA in the whole dataset. No imputation was necessary in the replication group, as all patients had detectable expression levels of the differentially expressed miRNAs. Gene-set enrichment analyses were conducted according to Subramanian et al [20]. Analysis of HNF1α and HNF1β binding sites in upstream regions of the analysed miRNAs was performed using the PROMO3 tool, linked to the TRANSFAC database [21].

## Results

**Fig. 1:**
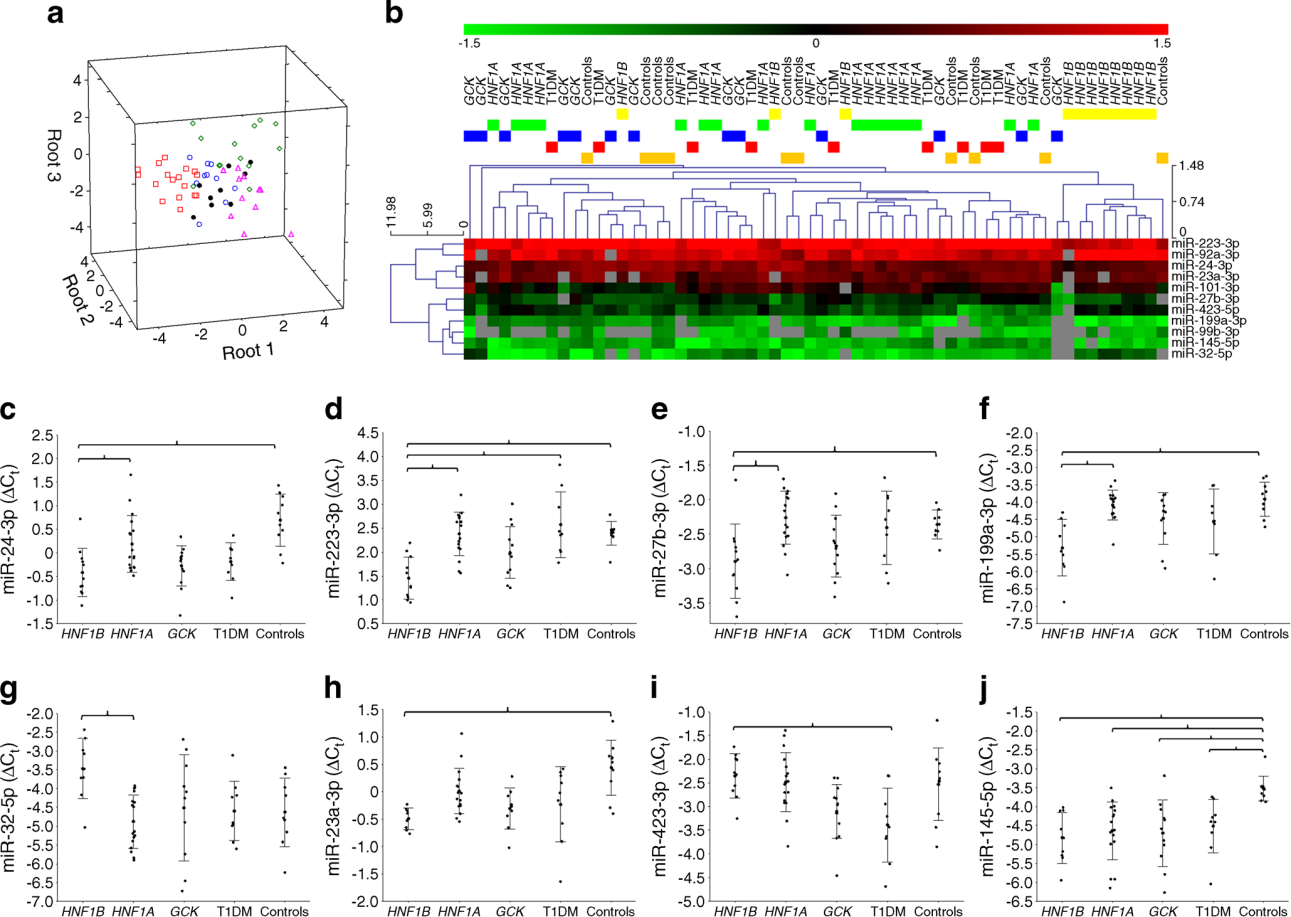
Serum miRNA profiling in the group of Polish patients with monogenic diabetes. (**a**) Linear discriminant analysis of miRNA profiles in the five compared groups. The axes denote the first three canonical roots. The five groups are represented by: *HNF1B*-MODY (pink triangles), *HNF1A*-MODY (red squares), *GCK*-MODY (green diamonds), type 1 diabetes (T1DM, black circles), controls (blue circles). (**b**) Hierarchical clustering of 11 miRNAs with significantly different expression levels between the five studied groups. Red denotes higher levels, green represents lower levels. Grey squares represent samples without detectable levels of a given miRNA. Expression levels of: (**c**) miR-24 ΔC_t_; (**d**) miR-223 ΔC_t_; (**e**) miR-27b ΔC_t_; (**f**) miR-199a ΔC_t_; (**g**) miR-32 ΔC_t_; (**h**) miR-23a ΔC_t_; (**i**) miR-423 ΔC_t_; (**j**) miR-145 ΔC_t_. Brackets are used to connect the groups with significant (*p* < 0.05) pairwise differences in post hoc comparisons between *HNF1B*-MODY and other groups. A detailed list of all pairwise post hoc tests is provided in ESM Table 3

The clinical characteristics of the primary group are presented in Table 1. No significant differences in sex and age distribution were noted between controls and all three MODY groups (*p* = 0.10 and *p* = 0.19, respectively). Patients with type 1 diabetes did not differ in HbA_1c_ level compared with those in the three MODY groups (*p* = 0.11). No significant differences were noted in BMI values (*p* = 0.10). Expression of the studied miRNAs, presented as ΔC_t_ values, is reported in ESM Table 2. A total of 83 miRNAs were present in at least five sera from each group (ESM Table 3). Linear discriminant analysis showed efficient discrimination between groups, using miRNA profiles with a potential for distinguishing *HNF1A*-MODY and *HNF1B*-MODY groups (Fig. 1a). Significance criterion was met by 11 distinct miRNAs: miR-223, miR-24, miR-99b, miR-423, miR-92a, miR-27b, miR-23a, miR-199a, miR-101, miR-145 and miR-32; these are presented on a hierarchical cluster heatmap in Fig. 1b. Among the 11 differentially expressed miRNAs (significant in ANOVA), eight differed significantly between *HNF1B*-MODY and at least one of the other groups (miR-32, miR-223, miR-23a, miR-199a, miR-27b, miR-24, miR-145 and miR-423; ESM Table 3). Expression levels (presented as ΔC_t_s) of those miRNAs are presented in Fig. 1c–j. The most striking differences were found between the *HNF1B*-MODY and *HNF1A*-MODY groups, evidenced by lower expression levels of miR-223, miR-24, miR-27b and miR-199a in the former. Only miR-32 showed higher expression in *HNF1B* than in *HNF1A*-MODY. Another notable finding was miR-145, which showed significantly higher expression in controls than in all other groups, hinting at its utility as a marker for hyperglycaemia or beta cell impairment. Significant differences between the *GCK*-MODY, type 1 diabetes and control groups were less numerous, with miR-24 showing higher expression in controls than in patients with type 1 diabetes (adjusted *p* = 0.0060); miR-24, along with miR-23a, miR-145 and miR-99b, also showed significantly lower expression levels in *GCK*-MODY than in controls (*p* = 0.0011, *p* = 0.0103, *p* = 0.0042 and *p* = 0.0236, respectively). No significant differences were noted between the *GCK*-MODY and type 1 diabetic groups (ESM Table 3).

**Table 1:** Clinical characteristics of the studied groups

Study group	*HNF1A*-MODY	*HNF1B*-MODY	*GCK*-MODY	Type 1 diabetes	Controls
Polish patients (primary group)^a^					
Sex, M/F	4/13	5/6	8/5	3/6	2/8
Age at examination, years	32.00 (21.00–49.00)	25.44 (13.44–37.81)	20.74 (18.14–24.38)	14.18 (9.79–16.33)	28.2 (23.04–30.06)
BMI, kg/m^2^	20.00 (18.70–23.80)	23.30 (19.60–24.81)	23.09 (21.83–24.65)	18.00 (16.10–19.20)	22.95 (21.88–26.72)
Duration of diabetes, years	11.00 (7.00–17.00)	1.01 (0.27–3.41)	1.16 (0.38–10.79)	0.50 (0.46–3.53)	NA
HbA_1c_, %	6.50 (5.30–8.70)	5.90 (5.00–6.30)	6.41 (6.30–6.50)	6.80 (6.40–10.50)	NA
HbA_1c_, mmol/mol	48 (34–72)	41 (31–45)	47 (45–48)	51 (46–91)	NA
UK patients (replication group)^b^					
Sex, M/F	7/11	9/3	4/6	3/8	1/9
Age at examination, years	30.50 (21.00–40.00)	37.50 (21.50–42.50)	21.00 (19.00–25.00)	17.00 (15.00–29.00)	26.00 (19.00–30.00)
BMI, kg/m^2^	22.40 (20.00–24.60)	23.00 (19.07–24.40)	22.80 (21.40–25.10)	21.60 (20.90–25.00)	24.09 (22.41–24.90)
Duration of diabetes, years	1.00 (0.00–18.00	4.00 (0.00–8.00)	1.00 (0.00–1.00)	2.00 (1.00–5.00)	NA
HbA_1c_, %	6.50 (5.80–6.90)	7.30 (6.50–7.70)	6.35 (6.20–6.70)	7.00 (6.40–9.60)	NA
HbA_1c_, mmol/mol	48 (40–52)	53 (48–61)	46 (44–50)	53 (46–81)	NA

Data are medians (25–75% percentile values)

^a^Polish patients (primary group): *HNF1A*-MODY (*n* = 17, from 12 families), *HNF1B*-MODY (*n* = 11, from six families), *GCK*-MODY (*n* = 13, from 13 families), type 1 diabetes (*n* = 9, from nine families), controls (*n* = 10, from ten families)

^b^UK patients (replication group): *HNF1A*-MODY (*n* = 18, from 18 families), *HNF1B*-MODY (*n* = 12, from 12 families), *GCK*-MODY (*n* = 10, from ten families), type 1 diabetes (*n* = 11, from 11 families), controls (*n* = 10, from ten families)

NA, not applicable

Having established which of the circulating miRNAs seem to be dependent on *HNF1A* or *HNF1B* defects, we set about replicating the findings in our UK group of patients with monogenic diabetes (Table 1). In this group, patients with type 1 diabetes were marginally younger than those in the other groups (*p* = 0.03 in ANOVA), but pairwise differences between any of the groups were not significant. Patients with *GCK*-MODY showed lower HbA_1c_ levels (*p* = 0.0314 in ANOVA), but no significant results were noted in post hoc comparisons. No differences for BMI (*p* = 0.6320) were noted. Females were significantly over-represented in the UK *HNF1B*-MODY group (*p* = 0.0305).

**Fig. 2:**
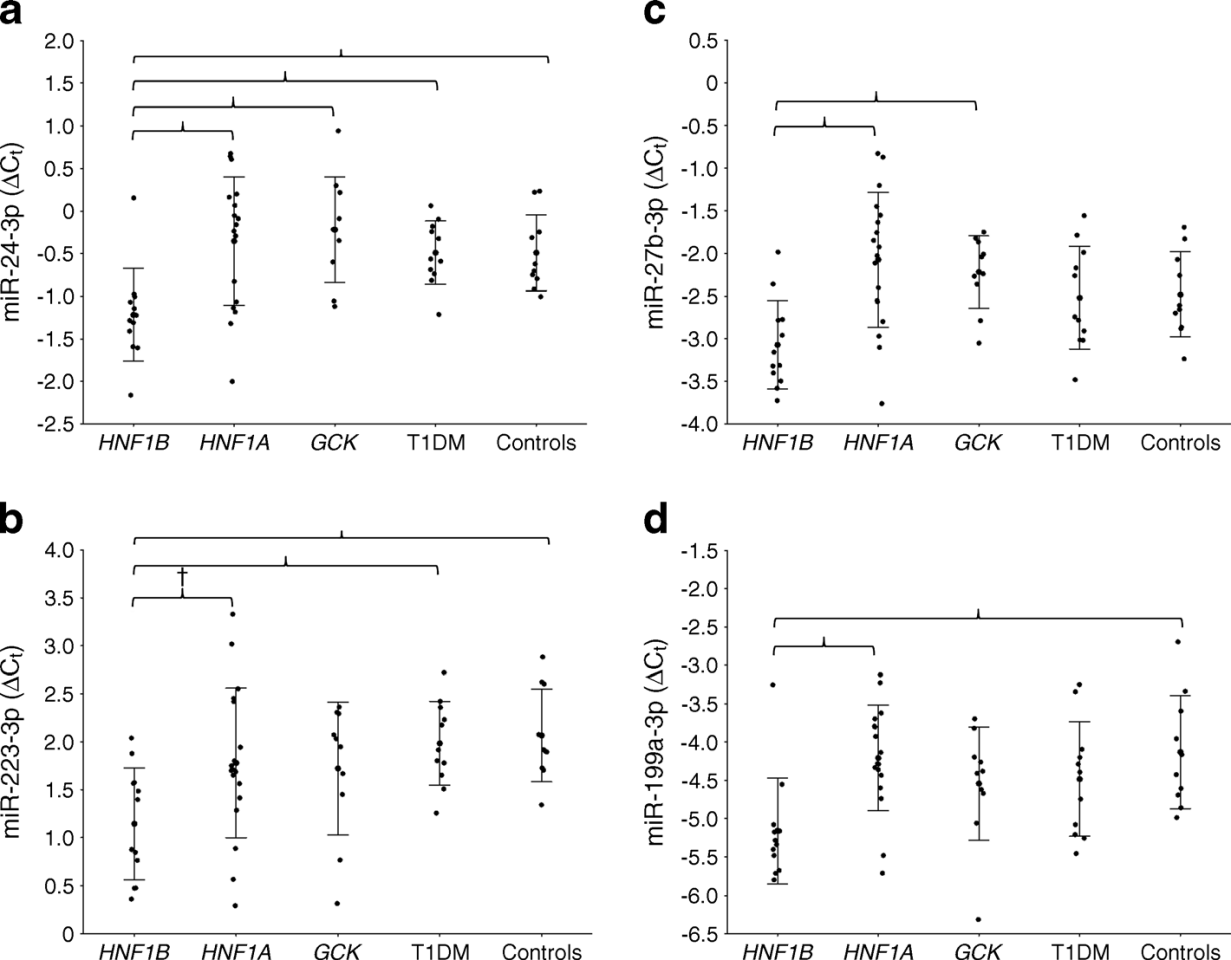
Comparisons of serum miRNA levels in the UK group: (**a**) miR-24; (**b**) miR-223; (**c**) miR-27b; (**d**) miR-199a. Brackets are used to connect the groups with significant (*p* < 0.05) pairwise differences; ^†^ *p* = 0.07; exact *p* values are shown in ESM Table 4

Within the UK group, five out of 11 differentially expressed miRNAs showed significant differences in ANOVA. In all cases, the *HNF1B*-MODY patients differed from at least one other group (ESM Table 4). The miRNAs with the most significant differences in expression levels mirrored those observed in the primary group: miR-24, miR-223, miR-27b and miR-199a (Fig. 2). Apart from miR-223, which correlated negatively with BMI in the type 1 diabetic and *HNF1A*-MODY groups, no significant correlations were noted for the clinical covariates with miRNA expression levels (ESM Table 5). Afterwards, we used ANCOVA models to determine whether the miRNA differences would persist after adjustment for age, sex, BMI and HbA_1c_. Significant differences in covariate-adjusted expression levels were noted for miR-24 (*p* = 0.0072), miR-223 (*p* = 0.0184), miR-27b (*p* = 0.0107) and miR-199a (*p* = 0.0435; ESM Fig. 1). In all cases, differences in post hoc tests were significant between the *HNF1A*- and *HNF1B*-MODY groups. A single patient with *HNF1B*-MODY aged ≤13 years was excluded from this analysis, as his raw BMI value would have been incomparable with the others. Adjusted expression of miR-423 did not differ significantly between the four groups of patients with diabetes (*p* = 0.3899), which excluded this miRNA from further analyses. A meta-analysis of effect sizes in the primary and replication groups performed to evaluate homogeneity of the observed differences showed convergent, significant results for miR-24, miR-223, miR-27b and miR-199a in both groups of patients (ESM Fig. 2).

**Fig. 3:**
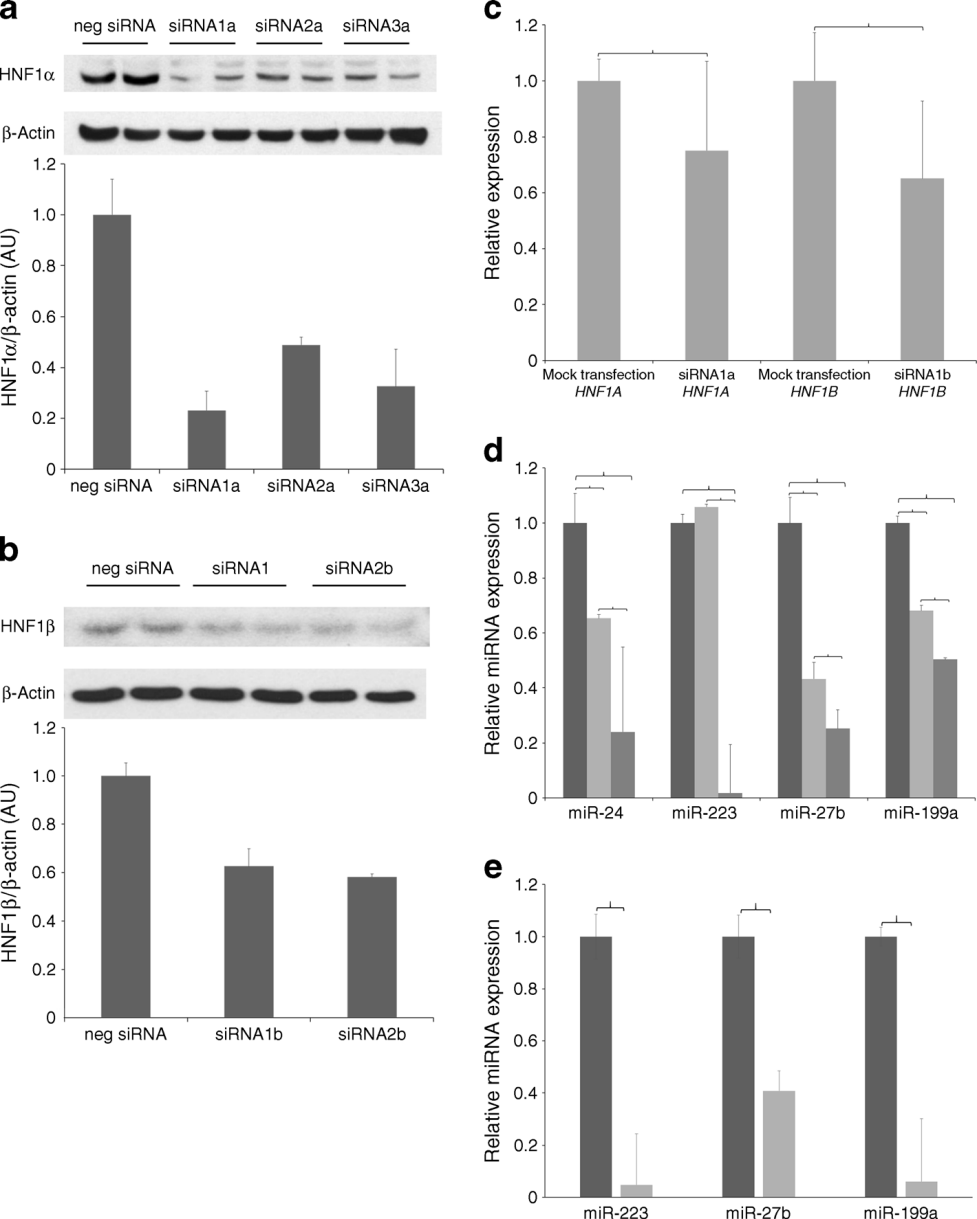
Impact of HNF1α or HNF1β knockdown on miRNA expression levels in human hepatocytes (HepG2). (**a**) Western blot analysis of siRNA-induced knockdown efficiency for HNF1α. (**b**) Western blot analysis of siRNA-induced knockdown efficiency for HNF1β. (**c**) mRNA expression levels of *HNF1A* and *HNF1B* after siRNA-induced gene silencing. (**d**) miRNA expression levels in HepG2 cells after mock nucleofection (leftmost columns in each block) after knockdown of HNF1α (middle columns) and HNF1β (rightmost columns). (**e**) miRNA expression levels measured in cell culture media after mock nucleofection (control group, dark grey) or HNF1β silencing (light grey); miR-24 was not detectable). Brackets are used to connect the groups with significant (*p* < 0.05) pairwise differences. AU, arbitrary units

Afterwards, we measured the impact of siRNA-induced knockdowns of HNF1α and HNF1β on the expression levels of miR-24, miR-223, miR-27b and miR-199a in human hepatocytes (HepG2). We tested three different siRNAs specific for HNF1α, which decreased HNF1α protein level by 80%, 52% and 63%, respectively (Fig. 3a), and two different siRNAs for HNF1β, which decreased the level of HNF1β by 40% and 42% in HepG2 cells (Fig. 3b). The reduced gene expression of *HNF1A* and *HNF1B* after siRNA transfections was also confirmed at the level of mRNA (Fig. 3c). For further experiments we used siRNA1a and siRNA1b, which showed the highest efficiency for downregulation of *HNF1A* and *HNF1B*, respectively. The silencing of *HNF1A* significantly decreased levels of miR-24, miR-27b and miR-199a, and had no effect on the miR-223 content in HepG2 cells (Fig. 3d). Downregulation of *HNF1B* in HepG2 cells resulted in significant reduction of all analysed miRNAs, with the highest impact on miR-223 whose level was reduced by almost 99%.

These data suggest that serum levels of miR-24, miR-223, miR-27b and miR-199a associated with *HNF1B* dysfunction might reflect changes in intracellular miRNA profile in the liver. To test this hypothesis, we tested the cell culture media for miRNA levels 48 h after *HNF1B* silencing in HepG2 cells. The levels of extracellular miR-223, miR-27b and miR-199a in HNF1β-deficient HepG2 cells were significantly reduced compared with controls (Fig. 3e). This suggests that hepatocytes are a likely source of circulating miRNAs responsible for an altered serum miRNA profile in patients with HNF-related monogenic diabetes.

**Fig. 4:**
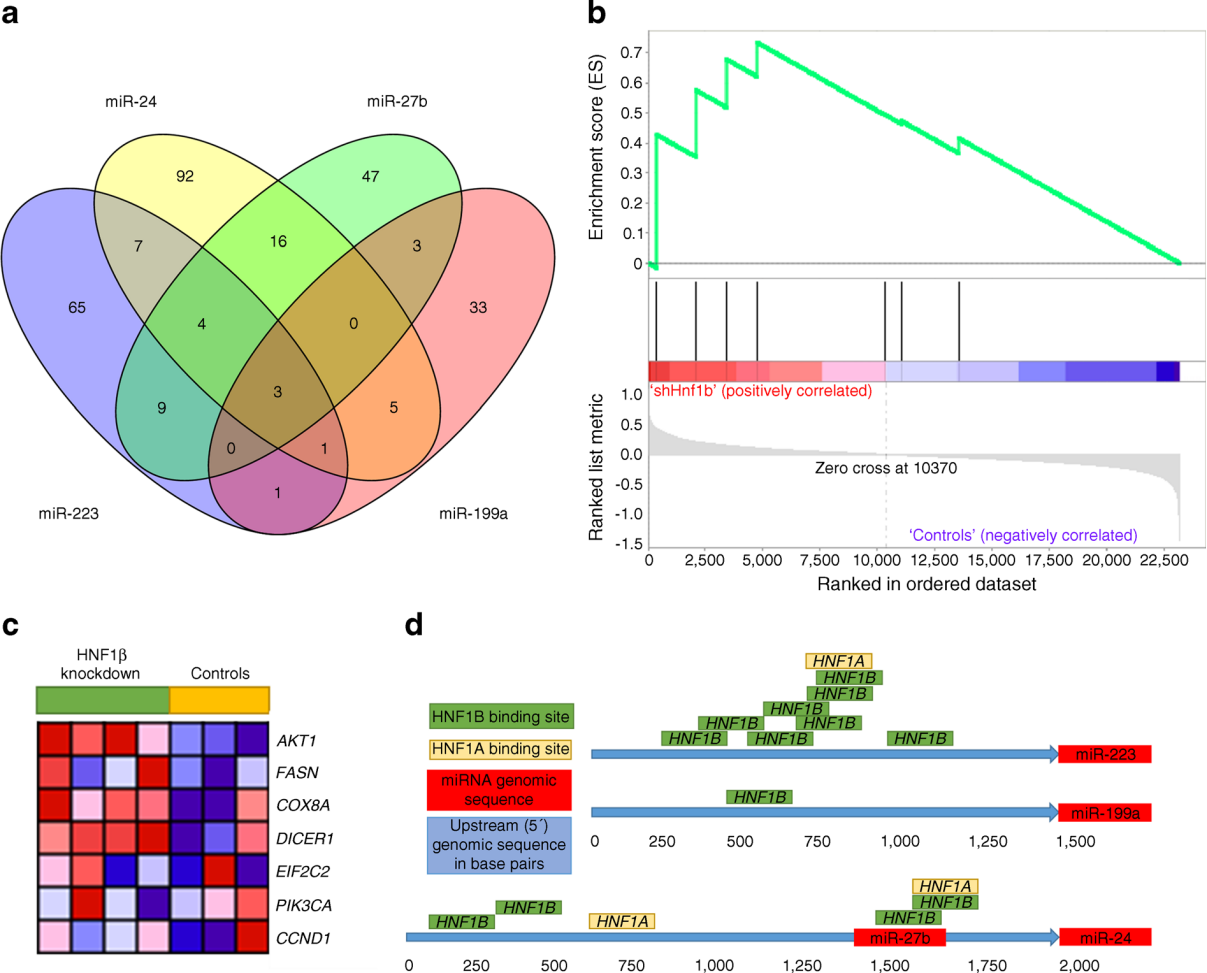
Biological targets of HNF1β-dependent miRNAs detectable in serum. (**a**) Venn diagram of genes with 3′UTR (untranslated region) fragments validated to be binding sites for the four miRNAs (according to the miRWalk database; ESM Table 6). (**b**) Enrichment plot from gene-set enrichment analysis showing the relative position of genes validated as targets of HNF1β-dependent miRNAs within the whole list of differentially regulated genes observed in the HNF1β knockdown gene expression experiment [9]. Green line, enrichment profile; vertical black lines, hits; grey lines and shading, ranking metric scores. The ranked list metric is shown as the signal-to-noise ratio. (**c**) Hierarchical clustering plot of gene expression. The first four samples are HNF1β knockdowns. (**d**) Schematic representation of the HNF1α and HNF1β binding sites located within the promoter regions of the four tested miRNAs. miR-27b and miR-24 are clustered together. Box sizes are not to scale. sh, Short hairpin

To ascertain potential pathways linked to the apparent downregulation of miRNA expression levels, we searched the miRWalk database [22] for validated targets of the four HNF1β-dependent miRNAs (Fig. 4a). A detailed list of genes with validated target sites of the four miRNAs and their combinations is provided in ESM Table 6. There are three genes to which all four miRNAs could potentially bind: *AKT1*, *CCND1* and *COX8A*. From these three, *CCND1* and *AKT1* were determined to be the most likely downstream effectors of the HNF1β signal through the miRNA network. To evaluate whether the potential target genes of the four miRNAs are downregulated in vivo, we performed gene-set enrichment analysis on an expression microarray dataset that describes gene expression in mice transfected with an adenovirus-borne silencer for *Hnf1b* (GSE42188 in Gene Expression Omnibus database [9]). miR-24, miR-223, miR-23 and miR-199a show 100% conservation of seed region sequences between humans and mice [23]. We composed a gene-set of eight target genes, all shown to have validated miRNA binding sites for at least three of the four HNF1β-dependent miRNAs: *Akt1*, *Fasn*, *Cox8a*, *Dicer1*, *Eif2c2* (also known as *Ago2*), *Pik3ca* and *Ccnd1* (ESM Table 6). This gene-set showed significant upregulation (*p* = 0.036) after *Hnf1b* silencing (Fig. 4b, c), thus supporting our claim that the downregulation of miRNAs due to loss of the HNF1β signal may result in upregulation of critical biological pathways. Details of the positions of the eight tested genes in GSEA (www.broad.mit.edu/gsea [20, 24], accessed 10 October 2015) are shown in ESM Table 7. Afterwards, using the TRANSFAC database, we screened 1,500 bp-long upstream genomic regions of the four miRNAs for binding sites of HNF1α and HNF1β. All four miRNAs had such sites (Fig. 4d, ESM Table 8). HNF1β binding sites were the most prevalent in the case of miR-223, which correlated with its having the greatest reduction in expression in the cell line experiments.

Finally, we examined the diagnostic potential of the four miRNAs. The cut-off values were calculated for the $\frac{\mathrm{miRNA}\;\mathrm{of} \operatorname {in}\mathrm{terest}}{\mathrm{average}\;\mathrm{of}\;\mathrm{three}\;\mathrm{reference}\;\mathrm{miRNAs}}$ ratios. All four miRNAs showed biomarker potential, with AUC values reaching or exceeding 0.8 (Table 2). We also analysed how the established cut-offs separated the *HNF1B*- from the *HNF1A*-MODY groups (Table 2). To finish, we created a multivariable logistic regression model using the four miRNAs in both primary and replication groups and examined their capability of separating *HNF1B*-MODY from patients with other types of diabetes (ESM Fig. 3a, b) or only from patients with *HNF1A*-MODY (ESM Fig. 3c, d). In all cases it was evident that in both cohorts the four validated miRNAs were differentially expressed in *HNF1A*- and *HNF1B*-MODY.

**Table 2:** Diagnostic efficacy of the evaluated miRNAs with *HNF1B*-MODY-dependent expression profile in the validation (UK) group. Cut-off values are ΔC_t_s calculated as listed in the Methods section. The last two columns represent the performance of the selected miRNAs in the primary group of Polish patients

Evaluated miRNA	Best cut-off value in the replication group	AUC (95% CI)	Sensitivity/specificity for best cut-off value (%)	AUC (95% CI) separating *HNF1B*- from *HNF1A*-MODY	Sensitivity/specificity for best cut-off value separating *HNF1B*- from *HNF1A*-MODY (%)
miR-24	<−0.97	0.86 (0.73, 0.99)	91.7 / 80.0	0.82 (0.66, 0.98)	91.7 / 72.2
miR-223	<1.57	0.79 (0.65, 0.92)	83.3 / 72.5	0.75 (0.56, 0.93)	83.3 / 66.7
miR-27b	<−2.77	0.83 (0.70, 0.96)	83.3 / 72.5	0.84 (0.70, 0.99)	83.3 / 77.8
miR-199a	<−5.07	0.82 (0.66, 0.97)	83.3 / 82.1	0.84 (0.67, 1.00)	83.3 / 88.2

## Discussion

Our results show that the levels of circulating miRNAs reflect the intracellular impact of a specific TF defect. Moreover, miRNAs that were identified as *HNF1B*-associated are likely to be involved in regulating metabolic functions associated with renal cyst formation.

That fact that miRNAs play an important part in the pathogenesis of diabetes is well established. Non-specific reduction of miRNA expression inhibits the formation of beta cells [25]. However, the question we addressed is whether specific miRNAs are linked to specific genetic causes of monogenic diabetes. An earlier study of miRNAs in monogenic diabetes caused by *HNF1A* mutations was performed by Bonner et al [26], who identified miRNAs linked to HNF1α knockdown in the insulin-1 cell model, and afterwards screened patients’ sera for those miRNAs. Our report partially confirms their findings, as we found serum miR-103 levels to be higher in the *HNF1A*-MODY group than in the controls and *HNF1B*-MODY groups, although the difference was not significant (*p* = 0.09; ESM Table 3).

We considered the possibility that at least some circulating miRNAs could originate from beta cells and reflect the remaining beta cell mass. However, given that none of the miRNAs tested in the primary group showed simultaneously significant differences between type 1 diabetes and *GCK*-MODY and between type 1 diabetes and control groups (ESM Table 3) we assumed that beta cells were not a likely source of the dysregulated miRNAs. Our assumption of extrapancreatic origin of MODY-associated miRNAs is further strengthened by a recent report by Latreille et al, who showed that beta cell secretion of miR-375 does not contribute significantly to its serum levels and does not differ between *HNF1A*-MODY and type 1 or type 2 diabetes [27]. Similarly, in our study the levels of miR-375 remained unaltered in both groups, confirming that the serum levels of this miRNA are probably not a good indicator of beta cell mass or function.

HNF1β is typically associated with pancreatic development [28], but high levels are also found in pancreatic islets, kidneys, gut and liver [29]. We thus examined HNF1β-deficient HepG2 human cell lines to investigate the link between HNF1α/HNF1β and miRNA expression. Silencing of *HNF1B* in human hepatocytes significantly decreased intracellular levels of miR-24, miR-27b, miR-199a and miR-223. Moreover, the reduced levels of these miRNAs were also found in the HepG2 culture media, indicating that extracellular transport of miRNA was also affected. Our observations do not negate the possibility that some miRNAs are associated with type 1 diabetes [30] and potentially with beta cell function [31], but rather show that the serum levels of miRNAs are probably a result of extrapancreatic effects, such as those presented in our hepatocyte experiment.

Interestingly, another study also showed that miR-223 and miR-199a are dependent on liver function [32], supporting our assumption that the most significant impact of an *HNF1A* or *HNF1B* defect would be manifested predominantly via hepatic function disruption rather than by beta cell-associated effects.

Linking miRNA and effector pathways in our study was based on bioinformatic predictions; however, a recent study on chromatin immunoprecipitation (ChIP) of HNF1β published by Hajarnis et al (deposited as GSE71250 in the Gene Expression Omnibus database) showed that miR-223 indeed has a strong HNF1β-binding site in its promoter region, while the intragenic miR-199a has an HNF1β-binding site in the promoter of the *dmm-2* gene [33]. For miR-27b and miR-24, a ChIP signal peak was also located upstream of the miRNA cluster. Human and mouse miRNAs show 100% homology of their seed region sequences, making both the bioinformatics prediction and ChIP data relevant to human data. Therefore, while it is possible that different regulatory mechanisms would determine miRNA expression in humans, it is very likely that the four miRNAs are regulated directly by HNF1β.

Our study does have several limitations linked mostly to the relative rarity of monogenic diabetes and specific recruitment protocols used in both participating countries. Since in Poland genetic screening for monogenic diabetes is performed predominantly from the paediatric diabetology perspective we rarely encounter a situation in which differentiation between type 2 and monogenic diabetes is an actual issue [4]. In our primary group, none of the MODY patients was obese, whereas in the UK one single patient with *HNF1B*-MODY crossed the 30 kg/m^2^ threshold of BMI. We therefore did not consider it feasible to include patients with type 2 diabetes, as comparison of miRNA profiles with such patients would be heavily confounded by age, BMI and treatment modality differences.

Second, treatment modalities or renal function variables could also be thought of as potential confounders. None of the patients in the *HNF1B*-MODY group had creatinine levels above 115 μmol/l, and none of the patients from other groups reported any symptoms concerning the urinary tract. However, it is possible that in patients with a longer duration of the disease, particularly *HNF1B*-MODY, renal insufficiency might play a role in shaping the miRNA transcription profile. Similarly we did not observe any differences in miRNA profiles in the MODY subgroups depending on treatment (data not shown), but this was most likely due to low statistical power.

Finally, the siRNA-induced suppression of *HNF1A* and *HNF1B* expression in the HepG2 cell line was lower than typically presented in knockdown studies (~60%). However, since homozygous mutations of *HNF1B* are lethal [34], we did not strive to suppress *HNF1B* completely and were satisfied with the ~60% suppression, which we deemed a situation similar to the heterozygous loss of *HNF1A* or *HNF1B* observed in our patients.

Monogenic diseases caused by loss of function of specific miRNAs were reported in haematology, where a miR-451-regulated locus is crucial for erythropoiesis [35]. An even more extreme example is auditory loss due to a single dominant point mutation of miR-96 [36]. Redundancy of miRNA targets protects against loss of a single miRNA, but some genes are uniquely regulated by only one miRNA, making single-miRNA diseases possible [37]. Whether point mutations of miRNA genes may be the next group of causative factors for monogenic diabetes requires further study.

In conclusion, we have shown that expression of the circulating miRNAs miR-24, miR-223, miR-27b and miR-199a depends on HNF1β function, making them potentially applicable in the diagnosis of *HNF1B*-MODY. The impact of HNF1β deficiency was also evidenced at cellular level, suggesting that these miRNAs may play a functional role in shaping the *HNF1B*-MODY phenotype.

## Electronic supplementary material

Below is the link to the electronic supplementary material.

ESM Methods (PDF 283 kb)

ESM Table 1 (PDF 185 kb)

ESM Table 2 (XLSX 239 kb)

ESM Table 3 (PDF 301 kb)

ESM Table 4 (PDF 99 kb)

ESM Table 5 (PDF 82 kb)

ESM Table 6 (PDF 398 kb)

ESM Table 7 (PDF 158 kb)

ESM Table 8 (PDF 172 kb)

ESM Fig. 1 (PDF 224 kb)

ESM Fig. 2 (PDF 320 kb)

ESM Fig. 3 (PDF 190 kb)

## Abbreviations

cDNA: Coding DNA
ChIP: Chromatin immunoprecipitation
GCK: Glucokinase
HNF1α: Hepatocyte nuclear factor 1 alpha
HNF1β: Hepatocyte nuclear factor 1 beta
miRNA: microRNA
qPCR: Quantitative real-time PCR
siRNA: Small interfering RNA
TF: Transcription factor
