# Supplementary material for: Differential regulation of serum microRNA expression by HNF1β and HNF1α transcription factors
Source: Diabetologia. 2016 Apr 8;59:1463–73. doi: 10.1007/s00125-016-3945-0 (PMC4901123; doi:10.1007/s00125-016-3945-0)
Supplement: Supplementary file 1 — (PDF 283 kb) [file 125_2016_3945_MOESM1_ESM.pdf]

Electronic Supplementary Material - Methods

Serum miRNA extraction, profiling and measurements

Prior to testing, all samples were stored at -80ºC and did not undergo more than two freeze-thaw cycles. Samples were shipped in dry ice to Exiqon (Copenhagen, Denmark) for miRNA extraction and profiling. Only miRNAs present in at least 5 samples from each of the five compared groups entered statistical analysis. Total RNA was extracted from serum using the Qiagen miRNeasy® Mini Kit (Qiagen, Hilden, Germany). Serum/plasma was thawed on ice and centrifuged at 3000 x g for 5 min in a 4°C microcentrifuge. An aliquot of 200 µL of serum/plasma per sample was transferred to a new microcentrifuge tube and 750 µL of a Qiazol Lysis Reagent (Qiagen, Hilden, Germany) mixture containing 1.25 µg/mL of MS2 bacteriophage RNA was added to the serum/plasma. The tube was mixed and incubated for 5 min followed by the addition of 200 µL chloroform. The tube was mixed, incubated for 2 min and centrifuged at 12,000 x g for 15 min in a 4°C microcentrifuge. The upper aqueous phase was transferred to a new microcentrifuge tube and 1.5 volumes of 100% ethanol were added. The contents were mixed thoroughly and 750 µL of the sample was transferred to a spin column in a collection tube followed by centrifugation at 15,000 x g for 30 sec at room temperature. The process was repeated until all remaining sample had been loaded. The column was rinsed with 700 µL Qiagen RWT buffer and centrifuged at 15,000 x g for 1 min at room temperature followed by another rinse with 500 µL Qiagen RPE buffer and centrifuged at 15,000 x g for 1 min at room temperature. A rinse step (500 µL Qiagen RPE buffer) was repeated twice. The Spin column was transferred to a new collection tube and centrifuged at 15,000 x g for 2 min at room temperature. The Spin column was transferred to a new microcentrifuge tube and the lid was left uncapped for 1 min to allow the column to dry. Total RNA was eluted by adding 50 µL of RNase-free water to the membrane of the Spin column and incubating for 1 min before centrifugation at 15,000 x g for 1 min at room temperature. The RNA was stored in a -80°C freezer. Each RNA sample was successfully reverse transcribed (RT) into cDNA and run on the miRCURY LNA™ Universal RT miRNA PCR Human panel I and II. For normalization of the data, we applied the average of the assays detected in all samples. A "no template" in the RT step was included as a negative control to detect RNA contamination in the RT step. Assays with 5 Cp's lower than the negative control were not included it in the data analysis. For assays that did not yield any signal on the negative control, the upper limit of detection was set to Cp =37. An RNA spike-in control (Sp6) was added in the reverse transcription reaction to evaluate the RT reaction. In addition to this a DNA spike-in (Sp3) was present in triplicate on all panels to test for contamination. To assess haemolysis, two miRNAs were used: miRNA-451, present in high levels within erythrocytes, and miRNA-23a, which is not affected by haemolysis. A volume of 15 µL of RNA was reverse transcribed in 75 µL reactions using the miRCURY LNA™ Universal RT miRNA PCR, polyadenylation and cDNA synthesis kit (Exiqon, Vedbæk,

Denmark). cDNA was diluted 50 x and assayed in 10 µL PCRs according to the protocol for miRCURY LNA™ Universal RT miRNA PCR; each miRNA was assayed once by qPCR on the miRNA Ready-to-Use PCR, Human panel I and panel II. Negative controls excluding template from the reverse transcription reaction were performed and profiled like the samples. The amplification was performed in a LightCycler® 480 Real-Time PCR System (Roche, Basel, Switzerland) in 384-well plates. The amplification curves were analysed using the Roche LC software to determine the threshold cycle (Ct) of the qPCR.

Samples from the replication group were analysed using Exiqon pick-and-mix real-time PCR arrays, composed for 11 differentially expressed miRNAs in the Polish group and an additional three miRNAs (miR-142-3p, miR-126-3p, miR-16-5p) with universal expression across samples and lack of significant differences between the compared groups of *HNF1A*-, *GCK*-MODY, T1DM and controls. All reactions were performed in triplicate using the same methodology and equipment as ones used in the profiling experiment of samples from the Polish group.

miRNA realtime qPCR assays in the replication group

Exiqon's miRCURY™ RNA Isolation Kit – Biofluids (cat. no. EQ-300112, Exiqon, Vedbæk, Denmark) was used to isolate total RNA including the miRNA fraction from 200 µL serum. To improve isolation, 1 µL of Exiqon RNA spike-in mix (cat. no. EQ-203203, Exiqon, Vedbæk, Denmark) and 2 µg glycogen, RNA grade (cat. no. R0551, Fermentas, Vilinius, Lithuania) as an RNA-free carrier were add. The miRCURY LNA™ Universal RT miRNA PCR System (cat. no. EQ-203301, Exiqon, Vedbæk, Denmark) was applied to synthesise cDNA. Each reverse transcription reaction included 4 µL 5x reaction buffer, 9 µL nuclease-free water, 2 µL enzyme mix, 1 µL synthetic RNA spike ins, 16 µL template total RNA in a total volume of 32 µL . The RT reaction was incubated for 60 min at 42°C, then for 5 min at 95°C to inactivate the reverse transcriptase, and then held at 4°C. The levels of 11 miRNAs were quantified by using real-time qPCR with the Mx3005P QPCR System (Agilent Technologies, Santa Clara, CA, USA). The total reaction volume of each qPCR was 10 µL – 4 µL cDNA (diluted 80x), 5 µL PCR ExiLENT SYBR Green master mix (cat. no. EQ-203421, Exiqon, Vedbæk, Denmark), 1 µL PCR primer set (cat. no. EQ-204227, EQ-204260, EQ-204291, EQ-204536, EQ-204734, EQ-204772, EQ-205702, EQ-205915, EQ-205986, EQ-204063, Exiqon, Vedbæk, Denmark). The qPCR with real-time detection was performed in a 96-well plate with the following conditions: polymerase activation/denaturation for 10 min at 95°C, and amplification consisting of 45 cycles of 10 sec at 95°C and 1 min at 60°C; ramp-rate was 1.6°C/sec). All reactions were run in triplicate.

Silencing of HNF1B and HNF1A genes – cell line experiment

Briefly, cells were trypsinised and $1x10^6$ cells were centrifuged at 200 x *g* for 10 min, and the pellet was suspended in 100 µL of Nucleofector Solution V. Next, the Nucleofector solution/cell pellet mixture was combined with 10-100 nM negative or target siRNA and then transferred into the cuvette provided by Lonza. The cuvette was placed inside the Nucleofector machine and cells were electroporated using programme T-028. After transfection, 500 µL of pre-warmed DMEM was added to the cuvette and the cells were immediately transferred into corresponding wells pre-filled with 1.5 mL of DMEM. Following transfection, the cells were put back into the humidified 37°C/5% CO_2 incubator until further use. Storing the cell suspension longer than 15 min in Nucleofector solution was avoided due to possible reduction of cell viability and gene transfer efficiency. Cells without siRNA were used as controls. After 48 hours of transfection, cells and culture media were harvested and stored for further analysis.

Expression of *HNF1A* and *HNF1B* and HNF-dependent miRNAs

Reverse transcription and HNF1A/B expression study

Each reverse transcriptase reaction contained 1000 ng of RNA (conc. 40 ng/µL), 5 µL of 10x RT Random Primers, 2.5 µL of MultiScribe™ Reverse Transcriptase, 2 µL of 25x dNTP mix (100 mM) and 5 µL of 10x RT Buffer in a total volume of 50 µL. The RT reaction was incubated for 5 sec at 72°C, 10 min at 25°C, 2.5 h at 37°C and then held at 4°C.

Each qPCR included: 25 ng of cDNA, 0.7 µL of each primer diluted to 10 µmol (HNF1a_F3cDNA – ID:167806, HNF1a_R3cDNA – ID:167807, HNF1b_F2cDNA – ID:167809, HNF1b_F1cDNA – ID:167808, Genomed, Warsaw, Poland) and 10 µL SYBR® Green PCR Master Mix (cat. no. 4309155, Applied Biosystems, Foster City, CA, USA) in a total volume of 15 µL. All reactions were run in triplicate, and beta-actin was used as an endogenous control. The real-time thermal profile was: 50°C for 2 min, 95°C for 10 min, followed by 40 cycles of 95°C for 30 sec and 60°C for 1 min.

Western blot analysis – study of HNF1A/B silencing

For Western blot analyses, cell pellets were collected and lysed for 30 min in ice-cold buffer (50 mM Tris-HCl, pH 7.4, 5 mM EDTA, 1% Triton X-100, 150 mM NaCl) containing protease (10 µg/µL leupeptin, 5 µg/µL pepstatin A, 2 µg/µL aprotinine and 1 mM phenylmethylsulfonyl fluoride), and phosphatase inhibitors (1 mM sodium orthovanadate, 10 mM sodium fluoride). After centrifugation at 10,000 x *g* and 4°C for 20 min, the supernatants were used as whole-cell lysates. Protein content was determined with the Bio-Rad Protein Assay (Bio-Rad, Hercules, CA, USA) using bovine serum albumin (BSA) as the standard. An equal amount of each protein sample was resolved by SDS-PAGE and transferred onto polyvinylidene difluoride membrane (Millipore, Billerica, MA, USA). After blocking with 5% non-fat milk, membranes

were probed with appropriate primary and HRP-conjugated secondary antibodies, respectively. Bands were visualised using Chemiluminescence HR Substrate reagent (Millipore, Billerica, MA, USA).

miRNA - reverse transcription and expression measurement in cell cultures and culture medium

Total RNA including the miRNA fraction was isolated from 350 µL of culture supernatant or HepG2 cell pellet (10^6 cells), using the mirVana™ PARIS™ Kit (Ambion, Austin, TX, USA). cDNA was synthesised according to TaqMan® MiRNA Reverse Transcription protocol (Applied Biosystems, Foster City, CA, USA). Each reverse transcriptase reaction contained 40 ng miRNA (conc. 8 ng/µL), 3 µL of 5× RT primer, 0.15 µL of 100 mM dNTP, 0.19 µL of RNase inhibitor, 50 U of reverse transcriptase and 1.5 µL of 10× RT buffer in a total volume of 15 µL. The RT reaction was incubated for 30 min at 16°C, 30 min at 42°C, 5 min at 85°C and then held at 4°C. miRNA levels were quantified using real-time qPCR with the Mx3005P QPCR System (Agilent Technologies, Santa Clara, CA, USA). The 18-µL qPCR volume included 3 µL RT product, 10 µL TaqMan® Gene Expression Master Mix (Applied Biosystems, Foster City, CA, USA), and 1 µM 20x TaqMan® miRNA Assays (assays IDs: 000399, 002441, 000408, 000409, 002109, 002148, 002304, 002295, 002844, 000564, 001821, Applied Biosystems, Foster City, CA, USA). The qPCR with real-time detection was performed in a 96-well plate with the following temperature profile: 50°C for 2 min, 95°C for 10 min, followed by 40 cycles of 95°C for 30 sec and 60°C for 1 min. All reactions were run in triplicate. U6 snRNA was used as an endogenous control (ID: 001973, Applied Biosystems, Foster City, CA, USA).
