# Supplementary material for: Differential regulation of serum microRNA expression by HNF1β and HNF1α transcription factors
Source: Diabetologia. 2016 Apr 8;59:1463–73. doi: 10.1007/s00125-016-3945-0 (PMC4901123; doi:10.1007/s00125-016-3945-0)
Supplement: Supplementary file 2 — (PDF 185 kb) [file 125_2016_3945_MOESM2_ESM.pdf]

Supplemental Table 1 – List of mutations present in patients with monogenic diabetes in the two groups: primary and replication.

Primary group			Replication group		
Group	Mutation	Aminoacid change / effect	Group	Mutation	Aminoacid change / effect
HNF1B-MODY	c.[434del]	L145fs	*HNF1B-MODY*	deletion	deletion (whole *HNF1B*)
HNF1B-MODY	deletion	deletion (whole *HNF1B*)	*HNF1B-MODY*	deletion	deletion (whole *HNF1B*)
HNF1B-MODY	deletion	deletion (whole *HNF1B*)	*HNF1B-MODY*	c.[511T>C]	W171R
HNF1B-MODY	deletion	deletion (whole *HNF1B*)	*HNF1B-MODY*	deletion	deletion (whole *HNF1B*)
HNF1B-MODY	deletion	deletion (whole *HNF1B*)	*HNF1B-MODY*	deletion	deletion (whole *HNF1B*)
HNF1B-MODY	c.[742C>T]	Q248X	*HNF1B-MODY*	c.[475C>G]	P159A
HNF1B-MODY	c.[742C>T]	Q248X	*HNF1B-MODY*	deletion	deletion (whole *HNF1B*)
HNF1B-MODY	c.[742C>T]	Q248X	*HNF1B-MODY*	c.[1235dup]	V413fs
HNF1B-MODY	c.[742C>T]	Q248X	*HNF1B-MODY*	c.[544+3_544+6delAAGT]	Q182fs
HNF1B-MODY	c.[742C>T]	Q248X	*HNF1B-MODY*	deletion	deletion (whole *HNF1B*)
HNF1B-MODY	deletion	deletion (whole *HNF1B*)	*HNF1B-MODY*	deletion	deletion (whole *HNF1B*)
HNF1A-MODY	c.[862G>A]	G288R	*HNF1B-MODY*	deletion	deletion (whole *HNF1B*)
HNF1A-MODY	c.[862G>A]	G288R	*HNF1A-MODY*	c.[392G>A]	R131Q
HNF1A-MODY	c.[811C>T]	R271W	*HNF1A-MODY*	c.[461T>C]	M154T
HNF1A-MODY	c.[197dup]	T67fs	*HNF1A-MODY*	c.[484C>G]	L162V

HNF1A-MODY	c.[511C>T]	R171X	*HNF1A-MODY*	c.[380_382del]	N127del
HNF1A-MODY	c.[1502-6G>A]	Splice site mutation IVS7	*HNF1A-MODY*	deletion	deletion (whole *HNF1A*)
HNF1A-MODY	c.[824A>T]	E275V	*HNF1A-MODY*	c.[347C>T]	A116V
HNF1A-MODY	c.[675del]	S225fs	*HNF1A-MODY*	c.[476G>A]	R159Q
HNF1A-MODY	c.[872dup]	G292fs	*HNF1A-MODY*	c.[872dup]	G292fs
HNF1A-MODY	c.[1380_1406del]	Gln460_Leu468del	*HNF1A-MODY*	c.[872dup]	G292fs
HNF1A-MODY	c.[675del]	S225fs	*HNF1A-MODY*	c.[1340C>T]	P447L
HNF1A-MODY	c.[197dup]	T67fs	*HNF1A-MODY*	c.[872dup]	G292fs
HNF1A-MODY	c.[788G>A]	R263H	*HNF1A-MODY*	c.[1556C>T]	P519L
HNF1A-MODY	c.[872dup]	G292fs	*HNF1A-MODY*	c.[872dup]	G292fs
HNF1A-MODY	c.[392G>A]	R131N	*HNF1A-MODY*	c.[815G>T]	R272L
HNF1A-MODY	c.[1340C>T]	P447L	*HNF1A-MODY*	c.[685C>T]	R229X
HNF1A-MODY	c.[872dup]	G292fs	*HNF1A-MODY*	c.[872dup]	G292fs
GCK-MODY	c.[617C>T]	T206M	*HNF1A-MODY*	c.[872dup]	G292fs
GCK-MODY	c.[131G>A]	G44D	*HNF1A-MODY*	c.[811C>T]	R271W
GCK-MODY	c.[660C>A]	C220X	*GCK-MODY*	c.[130G>A]	G44S
GCK-MODY	c.[1258A>G]	K420E	*GCK-MODY*	c.[1306A>T]	I436F
GCK-MODY	c.[683C>T]	T228M	*GCK-MODY*	c.[539A>G]	N180S
GCK-MODY	c.[904G>T]	V302L	*GCK-MODY*	c.[556C>T]	R186X
GCK-MODY	c.[370G>A]	D124N	*GCK-MODY*	c.[1318G>T]	E440X
GCK-MODY	c.[571C>T]	R191W	*GCK-MODY*	c.[458C>A]	P153H

GCK-MODY	c.[797T>C]	L266P	*GCK-MODY*	c.[1160C>T]	A387V
GCK-MODY	c.[1135G>C]	A379P	*GCK-MODY*	c.[540T>G]	N180K
GCK-MODY	c.[556C>T]	R186X	*GCK-MODY*	c.[312insGGAGACCAAAC]	H105fs
GCK-MODY	c.[497A>T]	N166I	*GCK-MODY*	c.[56T>A]	I19N
GCK-MODY	c.[1150G>A]	A384T			
