# Supplementary material for: Differential regulation of serum microRNA expression by HNF1β and HNF1α transcription factors
Source: Diabetologia. 2016 Apr 8;59:1463–73. doi: 10.1007/s00125-016-3945-0 (PMC4901123; doi:10.1007/s00125-016-3945-0)
Supplement: Supplementary file 4 — (PDF 301 kb) [file 125_2016_3945_MOESM4_ESM.pdf]

Supplemental Table 3 – Comparisons of miRNA expression levels between the five groups of the primary group. For miRNAs that showed significant differences in analysis of variance, pairwise comparisons were performed as detailed in the manuscript.

	HNF1B-MODY		HNF1A-MODY		GCK-MODY		T1DM		Controls		One way ANOVA	FDR
	Mean	SD	Mean	SD	Mean	SD	Mean	SD	Mean	SD	p level	
hsa-miR-223-3p	0.63	0.22	0.99	0.18	0.83	0.23	1.01	0.24	1.03	0.11	0.0000	0.0024
hsa-miR-24-3p	-0.16	0.22	0.09	0.25	-0.10	0.18	-0.07	0.17	0.30	0.25	0.0001	0.0024
hsa-miR-23a-3p	-0.20	0.08	0.01	0.17	-0.11	0.13	-0.10	0.28	0.19	0.22	0.0007	0.0174
hsa-miR-199a-3p	-2.13	0.28	-1.69	0.18	-1.83	0.23	-1.79	0.36	-1.68	0.22	0.0008	0.0174
hsa-miR-101-3p	-0.48	0.26	-0.32	0.26	-0.88	0.54	-0.59	0.21	-0.57	0.34	0.0019	0.0322
hsa-miR-27b-3p	-1.23	0.24	-0.94	0.15	-1.12	0.27	-0.96	0.21	-1.02	0.07	0.0034	0.0402
hsa-miR-145-5p	-1.99	0.21	-1.93	0.33	-1.94	0.34	-1.80	0.34	-1.52	0.13	0.0034	0.0402
hsa-miR-99b-5p	-1.78	0.28	-2.03	0.17	-2.03	0.18	-1.78	0.15	-1.56	0.32	0.0046	0.0475
hsa-miR-32-5p	-1.41	0.30	-2.02	0.26	-1.82	0.63	-1.83	0.33	-1.99	0.34	0.0057	0.0477
hsa-miR-423-5p	-0.97	0.22	-1.03	0.24	-1.30	0.26	-1.35	0.34	-1.09	0.32	0.0061	0.0477
hsa-miR-92a-3p	0.78	0.29	0.77	0.18	0.49	0.22	0.49	0.24	0.59	0.32	0.0063	0.0477
hsa-miR-320a	0.61	0.24	0.32	0.24	0.31	0.28	0.25	0.36	0.18	0.31	0.01	0.0694
hsa-miR-320b	0.38	0.23	0.08	0.25	0.22	0.28	0.17	0.35	-0.05	0.28	0.01	0.0735
hsa-miR-107	-0.79	0.22	-0.80	0.17	-0.70	0.27	-1.06	0.33	-0.67	0.30	0.01	0.0809
hsa-miR-103a-3p	-0.26	0.41	-0.19	0.25	-0.20	0.25	-0.38	0.25	0.08	0.18	0.02	0.0947
hsa-let-7g-5p	-0.88	0.38	-0.86	0.26	-0.84	0.12	-1.07	0.26	-0.64	0.21	0.02	0.0973
hsa-miR-484	-0.47	0.21	-0.78	0.23	-0.68	0.34	-0.71	0.18	-0.77	0.09	0.02	0.1023
hsa-miR-144-5p	-1.30	0.31	-1.79	0.36	-1.69	0.47	-1.61	0.28	-1.73	0.29	0.03	0.1251
hsa-miR-335-5p	-1.98	0.19	-1.75	0.20	-1.96	0.33	-1.87	0.23	-2.06	0.26	0.03	0.1251
hsa-miR-16-2-3p	-1.78	0.29	-2.12	0.32	-2.01	0.45	-2.02	0.38	-2.39	0.39	0.03	0.1251
hsa-let-7a-5p	-1.94	0.20	-2.16	0.27	-1.92	0.24	-2.21	0.25	-1.95	0.20	0.03	0.1322
hsa-miR-15b-5p	-0.86	0.38	-0.66	0.18	-0.56	0.17	-0.67	0.19	-0.63	0.18	0.04	0.1437
hsa-miR-122-5p	-0.90	0.82	-1.16	0.56	-1.65	0.42	-1.44	0.60	-1.08	0.37	0.04	0.1552
hsa-miR-27a-3p	-0.58	0.16	-0.31	0.25	-0.53	0.23	-0.44	0.33	-0.45	0.15	0.05	0.1619
hsa-miR-148a-3p	-1.74	0.17	-1.67	0.20	-1.60	0.31	-1.38	0.25	-1.62	0.18	0.05	0.1619

hsa-miR-19b-3p	0.07	0.23	-0.02	0.25	0.08	0.33	0.30	0.15	-0.01	0.28	0.05	0.1710
hsa-miR-451a	1.77	0.15	1.71	0.27	1.41	0.50	1.40	0.42	1.59	0.34	0.06	0.1874
hsa-miR-192-5p	-1.72	0.42	-2.07	0.37	-1.84	0.31	-1.81	0.20	-2.17	0.47	0.06	0.1874
hsa-miR-20a-5p	-0.20	0.23	0.00	0.15	-0.15	0.24	-0.08	0.12	-0.04	0.20	0.07	0.2113
hsa-let-7b-5p	-0.61	0.24	-0.63	0.19	-0.52	0.19	-0.76	0.21	-0.74	0.30	0.08	0.2175
hsa-miR-144-3p	-0.38	0.35	-0.26	0.33	-0.65	0.61	-0.58	0.25	-0.64	0.48	0.08	0.2175
hsa-miR-181a-5p	-1.34	0.12	-1.50	0.25	-1.46	0.46	-1.71	0.20	-1.41	0.33	0.08	0.2184
hsa-miR-486-5p	0.10	0.51	-0.12	0.34	-0.35	0.52	-0.07	0.31	-0.35	0.43	0.09	0.2312
hsa-miR-590-5p	-2.01	0.48	-1.77	0.34	-1.66	0.40	-1.53	0.13	-1.83	0.41	0.10	0.2348
hsa-miR-21-5p	0.10	0.23	0.25	0.15	0.07	0.17	0.16	0.28	0.20	0.17	0.12	0.2663
hsa-miR-150-5p	-0.20	0.28	-0.47	0.28	-0.51	0.33	-0.38	0.38	-0.56	0.39	0.12	0.2663
hsa-miR-502-3p	-2.32	0.18	-2.41	0.25	-2.49	0.41	-2.58	0.25	-2.18	0.20	0.12	0.2735
hsa-miR-151a-5p	-1.45	0.25	-1.36	0.23	-1.25	0.15	-1.47	0.21	-1.28	0.24	0.13	0.2922
hsa-miR-338-3p	-2.32	0.32	-2.35	0.28	-2.11	0.25	-2.23	0.26	-2.14	0.20	0.15	0.3182
hsa-let-7f-5p	-1.79	0.42	-1.69	0.38	-1.72	0.30	-1.90	0.39	-1.47	0.32	0.15	0.3182
hsa-miR-23b-3p	-1.19	0.20	-1.15	0.16	-1.15	0.14	-1.25	0.32	-1.01	0.21	0.17	0.3422
hsa-miR-30b-5p	-1.02	0.32	-1.17	0.23	-0.96	0.24	-1.00	0.24	-1.21	0.46	0.17	0.3422
hsa-miR-15b-3p	-2.41	0.24	-2.59	0.31	-2.28	0.42	-2.45	0.35	-2.73	0.25	0.19	0.3506
hsa-miR-140-5p	-2.33	0.25	-2.31	0.22	-2.20	0.25	-2.13	0.14	-2.05	0.36	0.19	0.3506
hsa-miR-26a-5p	-0.51	0.19	-0.73	0.33	-0.58	0.35	-0.58	0.19	-0.84	0.50	0.19	0.3506
hsa-let-7e-5p	-2.40	0.26	-2.40	0.31	-2.32	0.39	-2.47	0.19	-2.08	0.38	0.20	0.3668
hsa-miR-30c-5p	-0.91	0.28	-1.01	0.23	-0.86	0.25	-1.02	0.27	-0.81	0.27	0.21	0.3774
hsa-miR-30d-5p	-2.09	0.14	-2.33	0.27	-2.24	0.32	-2.22	0.36	-2.47	0.31	0.22	0.3844
hsa-miR-15a-5p	0.46	0.32	0.27	0.18	0.26	0.28	0.18	0.44	0.33	0.19	0.23	0.3844
hsa-miR-106a-5p	-0.50	0.21	-0.38	0.18	-0.32	0.12	-0.39	0.15	-0.41	0.22	0.23	0.3844
hsa-miR-93-5p	-0.78	0.35	-0.68	0.30	-0.61	0.32	-0.52	0.14	-0.55	0.20	0.24	0.3896
hsa-miR-342-3p	-1.18	0.22	-1.25	0.23	-1.37	0.20	-1.10	0.31	-1.31	0.40	0.25	0.4034
hsa-miR-125b-5p	-1.95	0.51	-2.29	0.26	-2.16	0.27	-2.03	0.31	-2.15	0.47	0.26	0.4034
hsa-miR-424-5p	-0.85	0.27	-0.72	0.42	-1.02	0.25	-0.82	0.56	-0.70	0.40	0.28	0.4324
hsa-miR-22-3p	-1.97	0.20	-2.24	0.28	-2.20	0.51	-2.32	0.47	-2.12	0.24	0.29	0.4324

hsa-miR-425-3p	-2.24	0.13	-2.35	0.18	-2.24	0.23	-2.44	0.37	-2.48	0.16	0.32	0.4681
hsa-miR-99a-5p	-1.72	0.48	-1.95	0.29	-2.02	0.35	-1.84	0.28	-1.92	0.16	0.33	0.4728
hsa-miR-125a-5p	-1.52	0.23	-1.34	0.36	-1.23	0.43	-1.38	0.34	-1.17	0.39	0.33	0.4728
hsa-miR-185-5p	-0.67	0.11	-0.50	0.21	-0.59	0.31	-0.59	0.14	-0.68	0.17	0.36	0.4944
hsa-miR-29a-3p	-1.87	0.24	-1.93	0.23	-2.03	0.28	-1.81	0.29	-1.82	0.32	0.36	0.4944
hsa-miR-532-3p	-2.10	0.29	-2.34	0.29	-2.26	0.32	-2.44	0.40	-2.30	0.36	0.39	0.5268
hsa-miR-425-5p	-1.34	0.24	-1.25	0.20	-1.31	0.31	-1.45	0.47	-1.22	0.19	0.39	0.5268
hsa-miR-25-3p	-1.15	0.37	-1.34	0.28	-1.25	0.58	-1.11	0.39	-1.40	0.27	0.41	0.5414
hsa-miR-151a-3p	-1.87	0.12	-1.67	0.34	-1.74	0.25	-1.82	0.40	-1.84	0.29	0.46	0.5986
hsa-miR-126-3p	0.52	0.43	0.42	0.23	0.46	0.30	0.28	0.22	0.41	0.26	0.47	0.6062
hsa-miR-26b-5p	-1.39	0.22	-1.44	0.26	-1.32	0.31	-1.54	0.33	-1.36	0.39	0.52	0.6525
hsa-miR-28-5p	-2.21	0.29	-2.12	0.34	-2.17	0.39	-2.36	0.21	-2.14	0.34	0.56	0.6977
hsa-miR-146a-5p	-1.84	0.25	-1.68	0.22	-1.80	0.18	-1.74	0.26	-1.75	0.33	0.57	0.6977
hsa-miR-374b-5p	-2.05	0.22	-2.06	0.29	-2.00	0.18	-2.18	0.28	-2.07	0.20	0.64	0.7756
hsa-miR-16-5p	0.49	0.36	0.19	0.49	0.21	0.59	0.35	0.78	0.27	0.55	0.68	0.8121
hsa-miR-382-5p	-2.44	0.23	-2.40	0.44	-2.26	0.47	-2.49	0.47	-2.25	0.12	0.71	0.8339
hsa-let-7d-3p	-1.30	0.31	-1.30	0.19	-1.29	0.41	-1.32	0.38	-1.14	0.25	0.73	0.8366
hsa-let-7i-5p	-1.51	0.20	-1.63	0.29	-1.58	0.41	-1.63	0.35	-1.68	0.20	0.77	0.8700
hsa-miR-142-5p	-2.18	0.36	-2.02	0.26	-2.13	0.37	-2.03	0.35	-2.06	0.31	0.81	0.8989
hsa-miR-375	-2.42	0.33	-2.38	0.34	-2.41	0.62	-2.46	0.33	-2.22	0.32	0.81	0.8989
hsa-miR-30e-5p	-2.40	0.07	-2.42	0.25	-2.24	0.55	-2.40	0.27	-2.41	0.20	0.85	0.9098
hsa-miR-29c-3p	-1.55	0.25	-1.54	0.21	-1.65	0.32	-1.58	0.33	-1.53	0.27	0.85	0.9098
hsa-miR-339-3p	-2.58	0.51	-2.42	0.38	-2.38	0.42	-2.43	0.19	-2.52	0.34	0.85	0.9098
hsa-miR-222-3p	-1.19	0.17	-1.28	0.18	-1.27	0.16	-1.26	0.21	-1.24	0.21	0.87	0.9161
hsa-miR-142-3p	0.01	0.28	0.09	0.33	0.12	0.37	0.06	0.29	0.15	0.44	0.90	0.9292
hsa-miR-423-3p	-1.56	0.37	-1.49	0.39	-1.47	0.26	-1.48	0.42	-1.41	0.52	0.94	0.9666
hsa-miR-409-3p	-2.38	0.59	-2.31	0.31	-2.39	0.30	-2.26	0.39	-2.28	0.47	0.97	0.9678
hsa-miR-148b-3p	-1.61	0.26	-1.61	0.30	-1.61	0.31	-1.62	0.40	-1.54	0.18	0.97	0.9678

Pairwise comparisons performed using the Tukey's HSD test										
	HNF1B vs HNF1A	HNF1B vs GCK	HNF1B vs T1DM	HNF1B vs Controls	HNF1A vs GCK	HNF1A vs T1DM	HNF1a vs Control	GCK vs T1DM	GCK vs Control	T1DM vs Control
hsa-miR-223-3p	0.0005	0.0903	0.0002	0.0008	0.2545	0.9190	1.0000	0.0959	0.3600	0.9311
hsa-miR-24-3p	0.0292	0.9625	0.8577	0.0002	0.1193	0.4039	0.1213	0.9961	0.0011	0.0060
hsa-miR-23a-3p	0.2019	0.9285	0.7876	0.0018	0.5873	0.7968	0.2465	0.9967	0.0103	0.0265
hsa-miR-199a-3p	0.0026	0.0682	0.1594	0.0005	0.6697	0.6440	0.9756	0.9993	0.3483	0.3266
hsa-miR-101-3p	0.7920	0.2145	0.9449	0.9955	0.0023	0.3454	0.5121	0.6265	0.3474	0.9961
hsa-miR-27b-3p	0.0107	0.7507	0.1363	0.0833	0.1592	0.9553	0.9893	0.6950	0.5532	0.9995
hsa-miR-145-5p	0.9817	0.9960	0.8863	0.0025	0.9997	0.9957	0.0073	0.9809	0.0042	0.0332
hsa-miR-99b-5p	0.6226	0.3820	0.9980	0.5170	0.9919	0.8004	0.0545	0.5622	0.0236	0.3570
hsa-miR-32-5p	0.0197	0.1391	0.0919	0.0800	0.9079	0.9700	0.9794	0.9997	0.9991	1.0000
hsa-miR-423-5p	0.9910	0.0831	0.0105	0.9708	0.1164	0.0337	0.9998	0.8814	0.2869	0.0503
hsa-miR-92a-3p	0.9982	0.1180	0.2041	0.3191	0.1768	0.3349	0.4926	0.9999	0.9839	0.9965
