# Supplementary material for: Differential regulation of serum microRNA expression by HNF1β and HNF1α transcription factors
Source: Diabetologia. 2016 Apr 8;59:1463–73. doi: 10.1007/s00125-016-3945-0 (PMC4901123; doi:10.1007/s00125-016-3945-0)
Supplement: Supplementary file 5 — (PDF 99 kb) [file 125_2016_3945_MOESM5_ESM.pdf]

Supplemental Table 4 – Results of miRNA serum expression levels comparisons in the replication group. For miRNAs that showed significant differences in analysis of variance, pairwise comparisons were performed as detailed in the manuscript.

	HNF1B-MODY		HNF1A-MODY		GCK-MODY		T1DM		Controls		One way ANOVA
	Mean	SD	Mean	SD	Mean	SD	Mean	SD	Mean	SD	p level
hsa-miR-24-3p	-1.2168	0.5434	-0.3524	0.7523	-0.2185	0.6231	-0.5104	0.3632	-0.4597	0.4631	0.0013
hsa-miR-27b-3p	-3.0707	0.5200	-2.0748	0.7896	-2.2192	0.4235	-2.5334	0.5757	-2.4548	0.5211	0.0013
hsa-miR-199a-3p	-5.1579	0.6872	-4.2073	0.6887	-4.5416	0.7393	-4.4381	0.7297	-4.1500	0.7803	0.0072
hsa-miR-223-3p	1.1452	0.5796	1.7802	0.7809	1.7221	0.6931	1.9776	0.4185	2.0857	0.5104	0.0090
hsa-miR-423-5p	-3.6705	1.2081	-4.1390	1.1182	-3.7774	1.1310	-4.5158	0.7790	-4.9097	0.6968	0.0337
hsa-miR-32-5p	-6.0916	1.0714	-6.4157	0.9996	-6.5166	1.1344	-6.2741	0.7720	-6.9002	0.4953	0.0815
hsa-miR-23a-3p	-0.7832	0.6108	-0.2941	0.6850	-0.2165	0.5826	-0.3801	0.2708	-0.3240	0.5021	0.2287
hsa-miR-145-5p	-5.6280	0.7066	-5.2175	0.7285	-5.4829	0.6960	-5.1897	0.7149	-5.0101	0.6934	0.2609
hsa-miR-92a-3p	-0.7954	1.1113	-0.9005	0.6040	-0.9763	0.5009	-0.8570	0.6334	-1.1656	0.4934	0.2931
hsa-miR-101-3p	-2.7717	0.7986	-2.5266	0.5275	-2.4914	0.4946	-2.6935	0.4435	-2.8576	0.3428	0.3069
hsa-miR-32-5p	-6.0916	1.0714	-6.4157	0.9996	-6.5166	1.1344	-6.2741	0.7720	-6.9002	0.4953	0.3301

Pairwise comparisons performed using the Tukey's HSD test

miRNA	HNF1B vs HNF1A	HNF1B vs GCK	HNF1B vs T1DM	HNF1B vs Controls	HNF1A vs GCK	HNF1A vs T1DM	HNF1a vs Control	GCK vs T1DM	GCK vs Control	T1DM vs Control
hsa-miR-24-3p	0.0023	0.0021	0.0358	0.0431	0.9783	0.9740	0.9770	0.8322	0.8443	1.0000
hsa-miR-27b-3p	0.0006	0.0164	0.2106	0.1755	0.9750	0.3339	0.4586	0.7966	0.8764	0.9999
hsa-miR-199a-3p	0.0076	0.2751	0.1734	0.0123	0.7675	0.8582	0.9989	0.9997	0.7025	0.7938
hsa-miR-223-3p	0.0679	0.2224	0.0197	0.0105	0.9994	0.9156	0.7793	0.8762	0.7402	0.9983
hsa-miR-423-5p	0.7344	0.9993	0.4129	0.0326	0.8970	0.9532	0.2420	0.6099	0.0790	0.7181
