# Supplementary material for: Differential regulation of serum microRNA expression by HNF1β and HNF1α transcription factors
Source: Diabetologia. 2016 Apr 8;59:1463–73. doi: 10.1007/s00125-016-3945-0 (PMC4901123; doi:10.1007/s00125-016-3945-0)
Supplement: Supplementary file 6 — (PDF 82 kb) [file 125_2016_3945_MOESM6_ESM.pdf]

Supplemental Table 5 – Univariate correlations of clinical variables with miRNA levels within the four groups of patients with diabetes.

Correlation	T1DM		GCK		HNF1A		HNF1B	
	R	p	R	p	R	p	R	p
Age & hsa-miR-223-3p	-0.39	0.2419	-0.18	0.6119	-0.41	0.0920	-0.33	0.2969
Age & hsa-miR-27b-3p	-0.08	0.8093	0.22	0.5410	0.17	0.5018	0.21	0.5128
Age & hsa-miR-199a-3p	0.13	0.7066	-0.31	0.3901	0.03	0.9107	-0.03	0.9312
Age & hsa-miR-24-3p	-0.14	0.6866	0.21	0.5643	0.03	0.9029	0.34	0.2756
HbA1c & hsa-miR-223-3p	0.00	1.0000	0.12	0.7499	0.35	0.1533	-0.05	0.8881
HbA1c & hsa-miR-27b-3p	-0.13	0.6959	0.14	0.6992	0.01	0.9675	-0.13	0.6876
HbA1c & hsa-miR-199a-3p	0.31	0.3567	0.01	0.9867	-0.05	0.8439	-0.33	0.2951
HbA1c & hsa-miR-24-3p	-0.25	0.4531	-0.12	0.7499	-0.02	0.9415	0.36	0.2534
BMI & hsa-miR-223-3p	-0.61	0.0467	0.10	0.7892	-0.61	0.0073	0.20	0.5405
BMI & hsa-miR-27b-3p	0.05	0.8944	0.12	0.7379	-0.24	0.3349	-0.10	0.7530
BMI & hsa-miR-199a-3p	-0.03	0.9366	0.11	0.7635	-0.06	0.8183	-0.07	0.8284
BMI & hsa-miR-24-3p	-0.02	0.9577	0.17	0.6383	-0.27	0.2801	-0.09	0.7863
