# Supplementary material for: Differential regulation of serum microRNA expression by HNF1β and HNF1α transcription factors
Source: Diabetologia. 2016 Apr 8;59:1463–73. doi: 10.1007/s00125-016-3945-0 (PMC4901123; doi:10.1007/s00125-016-3945-0)
Supplement: Supplementary file 7 — (PDF 398 kb) [file 125_2016_3945_MOESM7_ESM.pdf]

Supplemental Table 6 – Dataset with the number of validated miRNA binding sites of the four HNF1B-dependent miRNAs according to the miRWalk database.

Target Gene	hsa-miR-223	hsa-miR-24	hsa-miR-27b	hsa-miR-199a	Total number of miRNA binding sites	Number of miRNAs with potential to bind to specific gene
AKT1	2	2	1	6	11	4
COX8A	4	4	1	2	11	4
CCND1	3	2	1	2	8	4
DICER1	3	12	3	0	18	3
EIF2C2	1	4	1	0	6	3
PIK3CA	2	2	1	0	5	3
ELSPBP1	1	2	1	0	4	3
FASN	1	1	0	2	4	3
TGFB1	0	6	1	0	7	2
CDKN2A	0	4	2	0	6	2
HNF4A	0	4	0	2	6	2
AHSA1	0	4	0	1	5	2
MAPK14	0	4	0	1	5	2
MYC	1	4	0	0	5	2
NOTCH1	0	4	1	0	5	2
JUN	2	2	0	0	4	2
PI3	0	2	0	2	4	2
PTEN	0	0	1	3	4	2
TNF	2	0	2	0	4	2
AKT2	0	2	1	0	3	2
BRCA1	1	2	0	0	3	2
CEBPA	1	2	0	0	3	2
DDX20	0	2	1	0	3	2
E2F1	2	0	1	0	3	2
FOXO3	1	2	0	0	3	2
FOXP1	0	2	1	0	3	2
GEMIN4	0	2	1	0	3	2
HAND1	0	2	1	0	3	2

KIT	0	2	1	0	3	2
NFKB1	1	0	0	2	3	2
NOS2A	1	2	0	0	3	2
NOS3	0	2	1	0	3	2
NPAT	0	2	1	0	3	2
PPARGC1A	0	2	1	0	3	2
PPIG	0	0	1	2	3	2
RNASEN	1	0	2	0	3	2
RUNX1	2	0	1	0	3	2
SLC7A5	0	2	1	0	3	2
SYNE1	1	2	0	0	3	2
TBX5	0	2	1	0	3	2
TWIST1	0	2	0	1	3	2
VEGFA	0	2	1	0	3	2
BID	1	0	1	0	2	2
CSF3	1	0	1	0	2	2
IL1B	1	0	1	0	2	2
MEF2C	1	0	1	0	2	2
MET	0	0	1	1	2	2
NFYC	0	1	1	0	2	2
STMN1	1	0	1	0	2	2
CD4	6	0	0	0	6	1
HIF1A	0	0	0	6	6	1
SIRT1	0	0	0	6	6	1
SMAD2	0	6	0	0	6	1
ACVR1B	0	4	0	0	4	1
CDKN1B	0	4	0	0	4	1
KIAA0152	0	4	0	0	4	1
KRAS	0	4	0	0	4	1
PDGFB	0	4	0	0	4	1
TLR4	4	0	0	0	4	1
FRAP1	3	0	0	0	3	1
H2AFX	0	3	0	0	3	1

IKBKB	0	0	0	3	3	1
IL6	3	0	0	0	3	1
LMO2	3	0	0	0	3	1
ZAP70	3	0	0	0	3	1
ACTG1	0	2	0	0	2	1
AGTR1	0	2	0	0	2	1
ALOX5	0	0	0	2	2	1
ALOX5AP	0	0	0	2	2	1
AMELX	0	0	0	2	2	1
ANXA6	0	2	0	0	2	1
APPBP2	0	2	0	0	2	1
ARHGAP12	0	0	0	2	2	1
ARID4B	0	2	0	0	2	1
AURKB	0	2	0	0	2	1
B3GALNT1	0	2	0	0	2	1
BCL2	2	0	0	0	2	1
BHLHB5	0	2	0	0	2	1
C6orf134	0	2	0	0	2	1
CALB1	0	0	0	2	2	1
CCL7	0	2	0	0	2	1
CCNA2	0	2	0	0	2	1
CCNB1	0	2	0	0	2	1
CCNT2	0	2	0	0	2	1
CDC2	0	2	0	0	2	1
CDK4	0	2	0	0	2	1
CDKN1A	0	2	0	0	2	1
CEBPB	0	2	0	0	2	1
CLTC	0	2	0	0	2	1
CRYGC	0	0	0	2	2	1
CYP1B1	0	0	2	0	2	1
CYP2A6	0	0	0	2	2	1
DCXR	0	2	0	0	2	1
DDIT3	0	2	0	0	2	1

DDR1	0	0	0	2	2	1
DECR1	0	0	0	2	2	1
DMTF1	0	2	0	0	2	1
DYRK2	0	2	0	0	2	1
E2F2	0	2	0	0	2	1
EGFL7	0	0	0	2	2	1
EGFR	0	0	2	0	2	1
ELF4	0	2	0	0	2	1
ENAM	0	0	0	2	2	1
ESR1	0	2	0	0	2	1
EZH2	0	0	0	2	2	1
FAF1	0	2	0	0	2	1
FASLG	0	0	0	2	2	1
FBXW11	0	2	0	0	2	1
FBXW7	2	0	0	0	2	1
FEN1	0	2	0	0	2	1
FGF7	0	2	0	0	2	1
FLI1	0	2	0	0	2	1
FSTL1	0	2	0	0	2	1
FURIN	0	2	0	0	2	1
GEMIN5	0	2	0	0	2	1
GJA1	0	2	0	0	2	1
GPD1	0	2	0	0	2	1
GRIA2	0	2	0	0	2	1
HIPK3	0	2	0	0	2	1
HMOX1	0	2	0	0	2	1
HN1L	0	2	0	0	2	1
IGF1R	2	0	0	0	2	1
IKBKE	0	2	0	0	2	1
IL10	2	0	0	0	2	1
INSR	0	0	0	2	2	1
KLF5	0	2	0	0	2	1
LAMC2	0	2	0	0	2	1

LIN7C	0	0	0	2	2	1
MAP2K4	0	2	0	0	2	1
MAPKAPK5	0	2	0	0	2	1
MDM4	0	0	0	2	2	1
MPP5	0	0	0	2	2	1
MYLIP	0	2	0	0	2	1
MYOD1	0	2	0	0	2	1
MYOG	0	2	0	0	2	1
NEUROD1	0	0	0	2	2	1
NLRP3	2	0	0	0	2	1
NME1	0	2	0	0	2	1
NOL3	0	2	0	0	2	1
NPHS2	0	2	0	0	2	1
OCA2	0	2	0	0	2	1
PARP8	0	2	0	0	2	1
POLD1	0	2	0	0	2	1
PPARA	0	0	2	0	2	1
PPARG	0	0	2	0	2	1
PPM1D	0	2	0	0	2	1
PRKG1	0	0	0	2	2	1
PRNP	0	0	0	2	2	1
PVRL1	0	0	0	2	2	1
RND1	0	0	0	2	2	1
ROS1	0	2	0	0	2	1
RPE	0	2	0	0	2	1
RPIA	0	2	0	0	2	1
SCN5A	0	2	0	0	2	1
SIP1	0	2	0	0	2	1
SMC1A	0	2	0	0	2	1
SNF8	0	2	0	0	2	1
SOCS3	0	2	0	0	2	1
SOX2	2	0	0	0	2	1
SOX6	0	2	0	0	2	1

SOX9	0	0	0	2	2	1
ST14	0	0	2	0	2	1
STK24	0	2	0	0	2	1
STK3	0	2	0	0	2	1
TAC1	0	2	0	0	2	1
TAS2R13	0	2	0	0	2	1
TMEM184B	0	2	0	0	2	1
TMEM54	0	0	0	2	2	1
TMOD3	0	2	0	0	2	1
TOP1	0	2	0	0	2	1
TP53	0	2	0	0	2	1
TPPP3	0	2	0	0	2	1
TRIB3	0	2	0	0	2	1
TSHZ3	0	2	0	0	2	1
UCP2	0	2	0	0	2	1
UTRN	0	2	0	0	2	1
VSNL1	0	2	0	0	2	1
WT1	0	2	0	0	2	1
XYLT2	0	2	0	0	2	1
ZIC3	0	2	0	0	2	1
ZNF828	0	0	0	2	2	1
ACE2	1	0	0	0	1	1
ACVR2A	0	0	1	0	1	1
ADM	0	0	1	0	1	1
ADORA2B	0	0	1	0	1	1
AICDA	1	0	0	0	1	1
AIFM1	0	1	0	0	1	1
APRT	1	0	0	0	1	1
ARCN1	1	0	0	0	1	1
BBS9	1	0	0	0	1	1
BCL6	1	0	0	0	1	1
BCR	1	0	0	0	1	1
C9orf3	0	0	1	0	1	1

CAB39L	0	0	1	0	1	1
CACNA1C	1	0	0	0	1	1
CACNB1	1	0	0	0	1	1
CASP3	1	0	0	0	1	1
CASP4	1	0	0	0	1	1
CD19	1	0	0	0	1	1
CD36	1	0	0	0	1	1
CD44	0	0	0	1	1	1
CD8A	1	0	0	0	1	1
CHEK1	0	0	1	0	1	1
COL1A1	0	0	1	0	1	1
COL4A2	0	0	1	0	1	1
COL5A3	0	0	1	0	1	1
CREB1	1	0	0	0	1	1
CREBBP	0	0	1	0	1	1
CSF1	1	0	0	0	1	1
CTNNBIP1	0	0	1	0	1	1
CYP3A4	0	0	1	0	1	1
DGCR8	1	0	0	0	1	1
DNAJB7	0	0	1	0	1	1
DNMT1	0	0	1	0	1	1
DPYD	0	0	1	0	1	1
DUSP2	0	0	1	0	1	1
EPHA2	0	0	1	0	1	1
EPHB2	0	0	1	0	1	1
FADD	0	1	0	0	1	1
FH	1	0	0	0	1	1
FLT3	1	0	0	0	1	1
FOS	1	0	0	0	1	1
FOXO1	0	0	1	0	1	1
G3BP1	0	0	1	0	1	1
GPAM	1	0	0	0	1	1
HDAC4	0	0	1	0	1	1

HDAC9	0	0	1	0	1	1
HGF	0	0	1	0	1	1
HMGA2	1	0	0	0	1	1
HMGB1	1	0	0	0	1	1
HSP90AA1	1	0	0	0	1	1
HSP90B1	1	0	0	0	1	1
IFNG	1	0	0	0	1	1
IL17A	1	0	0	0	1	1
IL17D	1	0	0	0	1	1
IRAK2	1	0	0	0	1	1
IRF4	1	0	0	0	1	1
ITGA6	0	0	1	0	1	1
ITGAM	1	0	0	0	1	1
JAK2	1	0	0	0	1	1
KITLG	1	0	0	0	1	1
KLF4	1	0	0	0	1	1
LITAF	0	0	1	0	1	1
LYZ	1	0	0	0	1	1
MAPK1	0	0	0	1	1	1
MAPK8	0	0	0	1	1	1
MAPK9	0	0	0	1	1	1
MMP13	0	0	1	0	1	1
MMP14	0	0	1	0	1	1
MSTN	0	0	1	0	1	1
NODAL	1	0	0	0	1	1
NOTCH4	0	0	1	0	1	1
NTRK1	0	1	0	0	1	1
OAT	0	0	1	0	1	1
OPRM1	1	0	0	0	1	1
PAX3	0	0	1	0	1	1
PAX7	0	0	1	0	1	1
PCNA	1	0	0	0	1	1
PDLIM5	1	0	0	0	1	1

PDX1	0	0	1	0	1	1
PRB1	1	0	0	0	1	1
PRDM1	1	0	0	0	1	1
PROM1	1	0	0	0	1	1
PTPRC	1	0	0	0	1	1
RB1	0	0	1	0	1	1
RC3H1	1	0	0	0	1	1
RHOB	1	0	0	0	1	1
RNMT	0	0	0	1	1	1
RNPC3	0	0	1	0	1	1
RUNX1T1	1	0	0	0	1	1
RYBP	0	0	1	0	1	1
SARS2	1	0	0	0	1	1
SCD	1	0	0	0	1	1
SLC22A3	1	0	0	0	1	1
SLC2A4	1	0	0	0	1	1
SLITRK1	0	1	0	0	1	1
SMAD7	1	0	0	0	1	1
SOD2	1	0	0	0	1	1
SPI1	0	1	0	0	1	1
TCF3	1	0	0	0	1	1
TGFB3	0	0	1	0	1	1
TIMM8A	0	0	1	0	1	1
TPT1	0	0	1	0	1	1
VDR	0	0	1	0	1	1
ZBTB10	0	0	1	0	1	1
ZEB1	0	0	1	0	1	1
