# Supplementary material for: Differential regulation of serum microRNA expression by HNF1β and HNF1α transcription factors
Source: Diabetologia. 2016 Apr 8;59:1463–73. doi: 10.1007/s00125-016-3945-0 (PMC4901123; doi:10.1007/s00125-016-3945-0)
Supplement: Supplementary file 8 — (PDF 158 kb) [file 125_2016_3945_MOESM8_ESM.pdf]

Supplemental Table 7 – Gene set enrichment analysis (www.broad.mit.edu/gsea method referenced in citations 20 and 24 of the main text) results of the dataset published by Kornfeld et al. (GSE42188). This gene set was significantly up-regulated in the *HNF1B*-knockdown experiment described in the abovementioned paper.

NAME	PROBE	DESCRIPTION	RANK IN GENE LIST	RANK METRIC SCORE	RUNNING ES	CORE ENRICHMENT
row_0	*Akt1*	thymoma viral proto-oncogene 1	384	0.4274	0.4261	Yes
row_1	*Fasn*	fatty acid synthase	2089	0.2154	0.5757	Yes
row_2	*Cox8a*	cytochrome c oxidase, subunit VIIIa	3404	0.1540	0.6786	Yes
row_3	*Dicer1*	Dicer1, Dcr-1 homolog (Drosophila)	4735	0.1096	0.7347	Yes
row_4	*Eif2c2*	eukaryotic translation initiation factor 2C, 2	10301	0.0011	0.4956	No
row_5	*Pik3ca*	phosphatidylinositol 3-kinase, catalytic, alpha polypeptide	11032	-0.0095	0.4739	No
row_6	*Ccnd1*	cyclin D1	13532	-0.0483	0.4161	No
