# Supplementary material for: Differential regulation of serum microRNA expression by HNF1β and HNF1α transcription factors
Source: Diabetologia. 2016 Apr 8;59:1463–73. doi: 10.1007/s00125-016-3945-0 (PMC4901123; doi:10.1007/s00125-016-3945-0)
Supplement: Supplementary file 9 — (PDF 172 kb) [file 125_2016_3945_MOESM9_ESM.pdf]

Supplemental Table 8 – Locations of HNF1B and HNF1A binding sites upstream of the genomic locations of the four *HNF1B*-associated miRNAs. Genomic location is based on the GRCh37/hg19 assembly of the human genome. Genomic data for the 1500 base pair-long upstream fragment were extracted for miR-223 and imR-199a. For miR-27b and miR-24, due to their proximity within the genome, both were extracted as a single overlapping sequence with an additional 1500 base pair fragment upstream of the miR-27b.

miR-223							
Sequence name	Factor name	Start position	End position	Dissimilarity	String	RE equally	RE query
chrX:65237512-65238821	HNF-1B [T01950]	250	258	12.60	AAAATAACC	0.09	0.08
chrX:65237512-65238821	HNF-1B [T01950]	356	364	9.31	TTTATAACC	0.06	0.06
chrX:65237512-65238821	HNF-1B [T01950]	561	569	12.82	TGTTATGTA	0.12	0.10
chrX:65237512-65238821	HNF-1B [T01950]	618	626	13.42	ACAATAACC	0.15	0.12
chrX:65237512-65238821	HNF-1B [T01950]	710	718	12.51	TGCCTAACT	0.09	0.08
chrX:65237512-65238821	HNF-1B [T01950]	749	757	6.71	TGTTTAACC	0.03	0.03
chrX:65237512-65238821	HNF-1B [T01950]	770	778	13.44	TGCATAACC	0.15	0.12
chrX:65237512-65238821	HNF-1B [T01950]	1031	1039	12.61	GGTTAGGAA	0.09	0.08
chrX:65237512-65238821	HNF-1A [T00368]	749	756	0.43	TGTTTAAC	0.16	0.15
miR-199a							
Sequence name	Factor name	Start position	End position	Dissimilarity	String	RE equally	RE query
chr19:10815926-10817496	HNF-1B [T01950]	507	515	14.87	AGTTACTTG	0.07	0.03
miR-27 and miR-24							
Sequence name	Factor name	Start position	End position	Dissimilarity	String	RE equally	RE query
chr9:97846234-97848370	HNF-1B [T01950]	71	79	10.34	TCTCTAACA	0.09	0.09
chr9:97846234-97848370	HNF-1B [T01950]	310	318	13.13	TGTGTAACG	0.20	0.20
chr9:97846234-97848370	HNF-1B [T01950]	1496	1504	10.34	TCTCTAACA	0.09	0.09
chr9:97846234-97848370	HNF-1B [T01950]	1600	1608	14.85	AGTTAAGTT	0.09	0.09
chr9:97846234-97848370	HNF-1A [T00368]	605	612	0.29	GTTAAAGT	0.26	0.27
chr9:97846234-97848370	HNF-1A [T00368]	1601	1608	4.83	GTTAAGTT	0.39	0.40
