# Supplementary material for: Differential regulation of serum microRNA expression by HNF1β and HNF1α transcription factors
Source: Diabetologia. 2016 Apr 8;59:1463–73. doi: 10.1007/s00125-016-3945-0 (PMC4901123; doi:10.1007/s00125-016-3945-0)

Supplemental figure 1 – Comparisons of serum miRNA expression values in the UK group (a – miR-24, b – miR-223, c – miR-27b, d – miR-199a) after adjustment for clinical covariates: age, sex, BMI and glycated haemoglobin level (e – miR-24, f – miR-223, g – miR-27b, h – miR-199a). Significant ($p<0.05$) pairwise comparisons are marked with brackets and exact p values. All p values for pairwise comparisons are shown in ESM table 4.

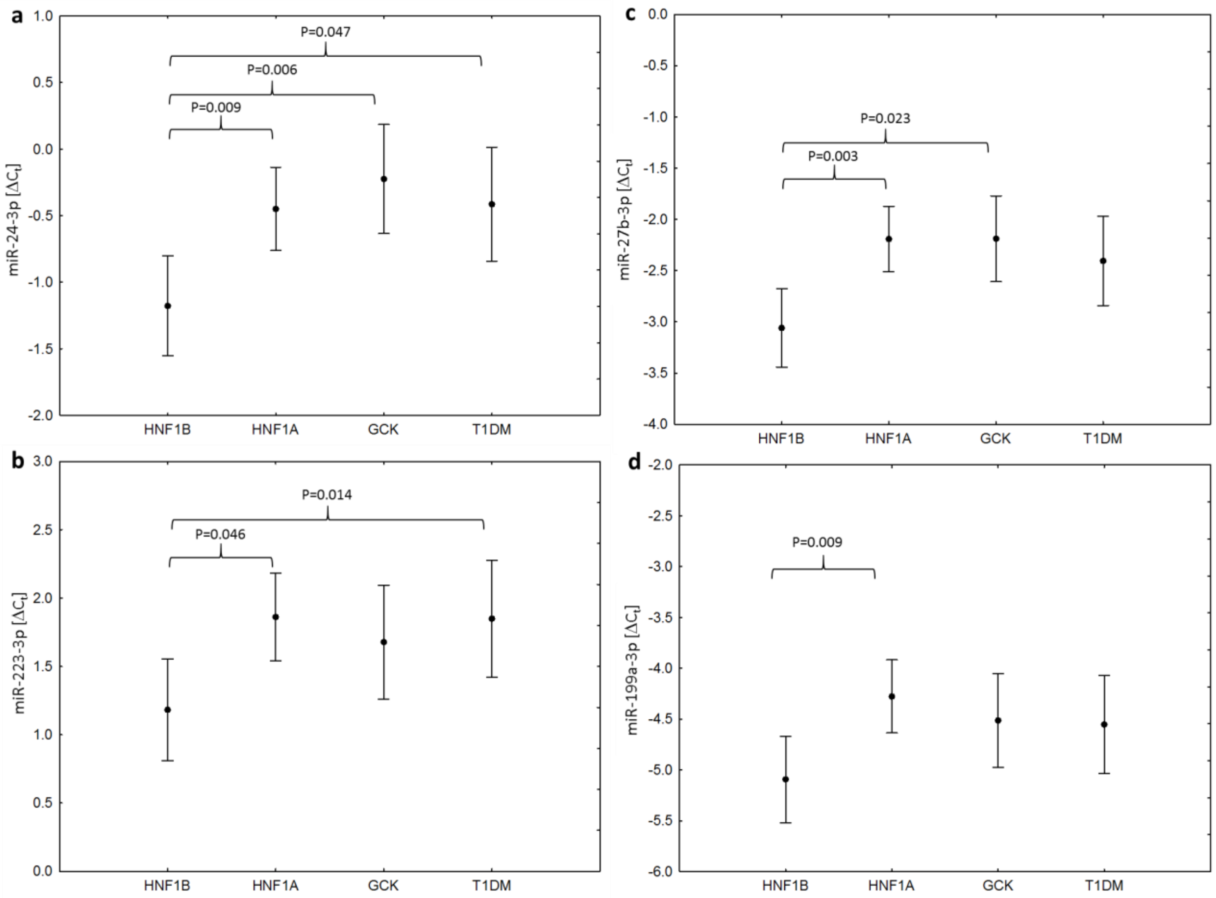

Supplement: Supplementary file 10 — (PDF 224 kb) [file 125_2016_3945_MOESM10_ESM.pdf]
