# Supplementary material for: Differential regulation of serum microRNA expression by HNF1β and HNF1α transcription factors
Source: Diabetologia. 2016 Apr 8;59:1463–73. doi: 10.1007/s00125-016-3945-0 (PMC4901123; doi:10.1007/s00125-016-3945-0)

Supplemental figure 2 – Metaanalysis of expression differences between the primary and replication groups. A) miR-24 differences between the two MODY types were similar in the two groups, results were highly homogeneous (Q=0.004; I^2<1%), b) miR-223 differences between the two MODY types were similar in the two groups, results showed moderate heterogeneity (Q=2.32; I^2=57%), c) miR-199a differences between the two HNF1-MODY groups were similar in the two groups, results showed moderate heterogeneity (Q=1.65; I^2=39.5%), d) miR-27b differences between the two HNF1A-MODY groups were similar in the two groups, results were highly homogeneous (Q=0.15; I^2<1%). e) the differences of miR-32 expression between the two HNF1-MODY were detected only in the Polish group, results were highly homogeneous (Q=9.32; I^2=86%).

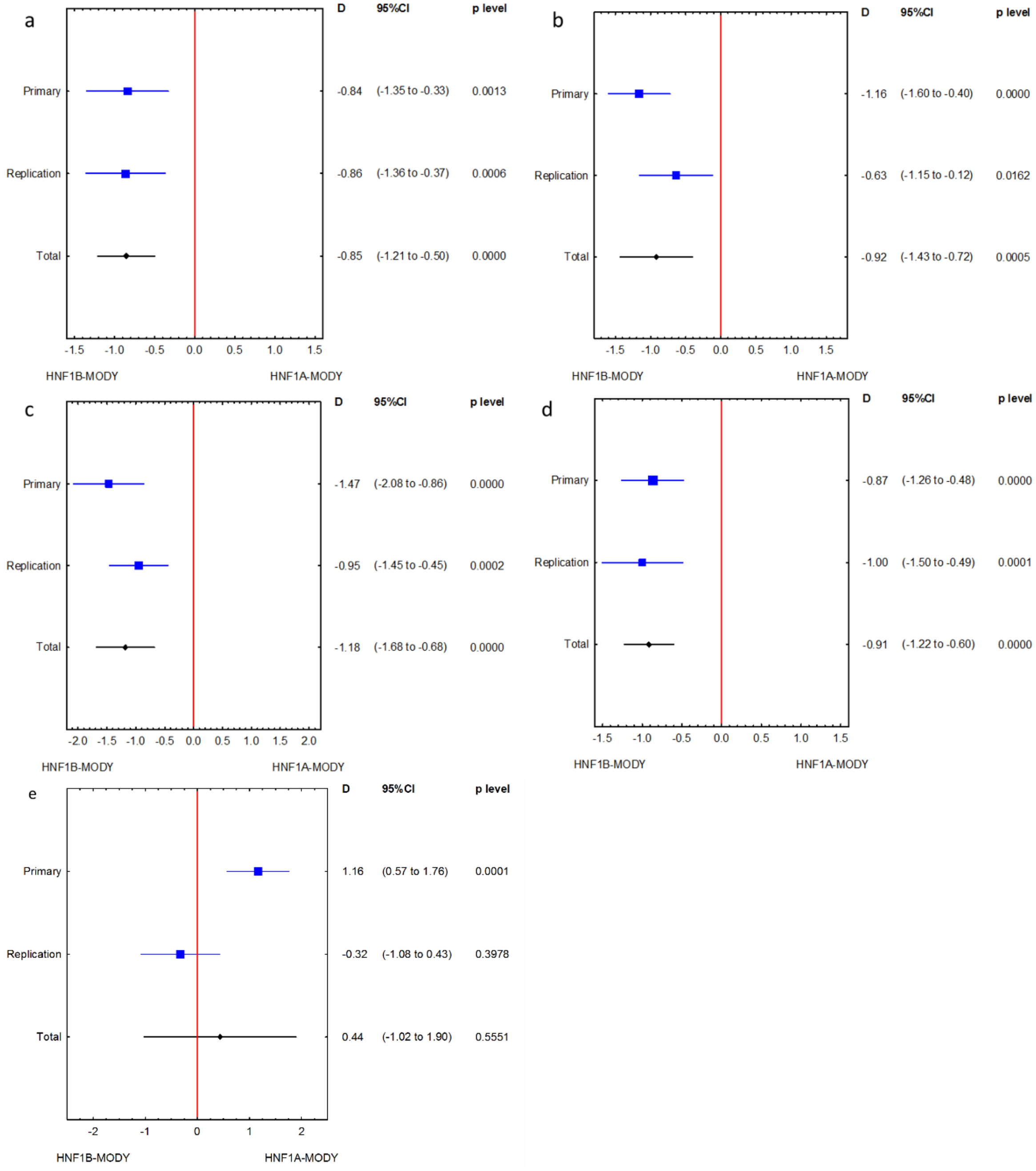

Supplement: Supplementary file 11 — (PDF 320 kb) [file 125_2016_3945_MOESM11_ESM.pdf]
