# Supplementary material for: Differential regulation of serum microRNA expression by HNF1β and HNF1α transcription factors
Source: Diabetologia. 2016 Apr 8;59:1463–73. doi: 10.1007/s00125-016-3945-0 (PMC4901123; doi:10.1007/s00125-016-3945-0)

Supplemental figure 3 – ROC curves of the multivariable model for separating *HNF1B*-MODY from: other patients with diabetes in the primary group (AUC=0.90 (95%CI:0.81-0.99)) (a), other patients with diabetes in the replication group (AUC=0.88 (95%CI:0.77-0.99) (b), ROC of the multivariable model for separating *HNF1B* from *HNF1A*-MODY in the primary (AUC=1.00 (c) and replication group (AUC=0.90 95%CI 0.78-1.00) (d).

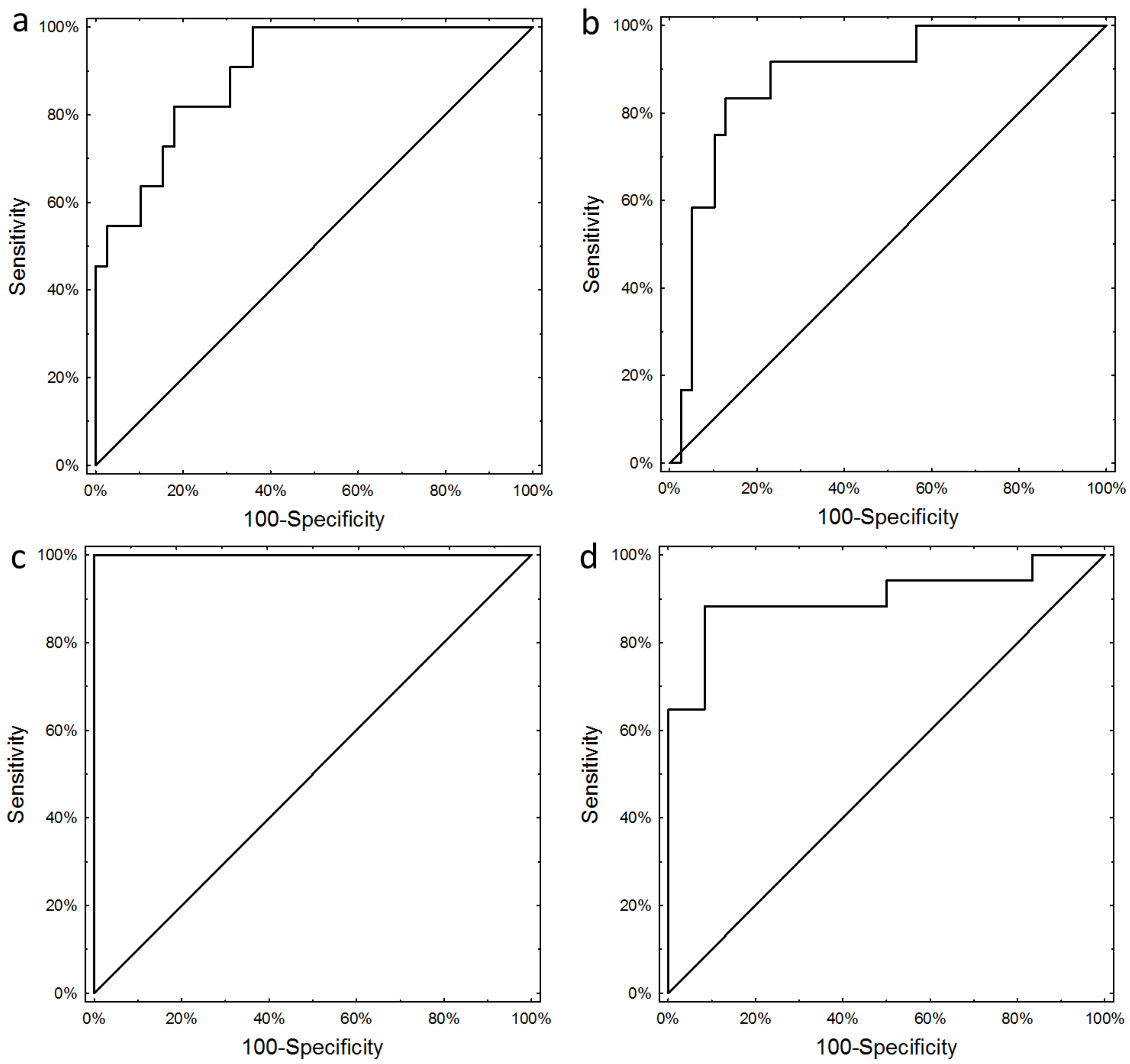

Supplement: Supplementary file 12 — (PDF 190 kb) [file 125_2016_3945_MOESM12_ESM.pdf]
